# Challenges and Good Practices in Preprocessing and Normalization of Untargeted DNA Adductomics Data in Exposomics Research

**DOI:** 10.1021/acs.analchem.5c06549

**Published:** 2026-03-16

**Authors:** Pablo Vangeenderhuysen, Matthijs Vynck, Liesa Engelen, Adrian Covaci, Tim Nawrot, Trancizeo Lipenga, Roger Pero-Gascon, Sarah De Saeger, Marthe De Boevre, Valerie McCormack, Lynn Vanhaecke, Lieselot Y. Hemeryck

**Affiliations:** † Laboratory of Integrative Metabolomics (LIMET), 26656 Ghent University, 9820 Merelbeke, Belgium; ‡ Centre for Environmental Sciences, 54496 Hasselt University, 3590 Diepenbeek, Belgium; § Toxicological Centre, University of Antwerp, 2610 Wilrijk, Belgium; ∥ Department of Public Health & Primary Care, Occupational & Environmental Medicine, KU Leuven, 3000 Leuven, Belgium; ⊥ Department of Bioanalysis, Centre of Excellence in Mycotoxicology and Public Health, Ghent University, 9000 Ghent, Belgium; # Department of Biomedical Sciences, Mzuzu University, Luwinga, P/BAG 201 Mzuzu, Malawi; ∇ Department of Chemical Engineering and Analytical Chemistry, Institute for Research on Nutrition and Food Safety (INSA-UB), University of Barcelona, 08007 Barcelona, Spain; ○ Department of Biotechnology and Food Technology, Faculty of Science, University of Johannesburg, Doornfontein Campus, Gauteng, 2094 Johannesburg, South Africa; ◆ Environment and Lifestyle Epidemiology Branch, International Agency for Research on Cancer (WHO-IARC), 69007 Lyon, France; ¶ Institute for Global Food Security, Queen’s University Belfast, BT7 1NN Belfast, U.K.

## Abstract

DNA adductomics is the study of the whole of DNA adducts in a biological sample and is a valuable asset to exposomics research. To date, a clear view on how to analyze larger sample series is lacking in DNA adductomics, and the preprocessing of untargeted DNA adductomics data is seldom applied. This work aimed to optimize a DNA adductomics data preprocessing workflow (in true untargeted mode). Building upon the xcms R package, we optimized parameters for peak detection, retention time alignment, and peak grouping to reliably detect and integrate putative DNA adduct LC-MS peaks. Next, to ensure reliable downstream data analysis, six sample- and feature-based normalization methods were tested and quantitatively evaluated in two data sets (placental tissue, *n* = 375, and blood samples, *n* = 51). As a result, a successful and reproducible procedure for optimization of xcms parameters for DNA adductomics is proposed. Furthermore, evaluation of normalization methods demonstrated the importance and limitations of objective (RSD* and D-ratio) and subjective, i.e., visual (PCA score plot) evaluation. This work supports reproducible and transparent untargeted DNA adductomics data preprocessing to be implemented in large-scale exposomics studies.

## Graphical abstract

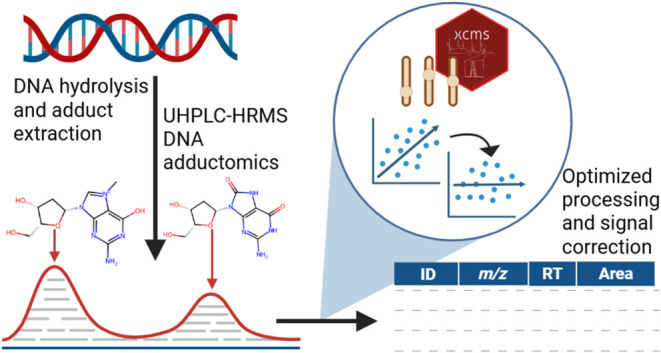

## Introduction

Since the exposome was defined by Wild,^1^ the influence of environmental exposures on biological systems has been widely recognized by the scientific community, leading to the development and rapid expansion of the exposomics research field. Exposomics aims to capture the comprehensive and cumulative effects of physical, chemical, biological, and psychosocial factors, collectively representing nongenetic drivers of health, throughout an individual’s life.^2,3^ Exposure to genotoxic chemicals can lead to modifications in human DNA, known as DNA adducts, which, if not repaired, can play a key role in carcinogenesis.^4^ Several analytical methods are available to study DNA adducts: ^32^P-postlabeling, liquid chromatography-, gas chromatography - mass spectrometry (LC-MS, GC-MS), LC-fluorescence, immunoassays and electrochemical detection.^5^ LC-MS has however become the method of choice for identification and quantitation of DNA adducts.^6–9^ Whereas other methods focus on the investigation of a smaller number of anticipated DNA adducts - depending on the research’s context (targeted analysis) - LC-MS­(/MS)-based DNA adductomics allows to screen for and analyze both known and unknown DNA adducts.^6,10,11^

The field of DNA adductomics is an emerging field of research, and while several analytical methods have been developed and validated, research toward and development of specific data preprocessing strategies are lagging behind.^6,7,10,11^ Ease of data analysis has been reported as one of the main challenges in the future of DNA adductomics.^11^ Indeed, recent advances in untargeted preprocessing^12,13^ of LC-MS data have focused on the requirements for metabolomics, lipidomics and proteomics, but seldomly take into account the specific intricacies related to DNA adductomics, such as the chemical complexity of samples in which to detect low levels of DNA adducts.^7,11,14^ To facilitate the identification of DNA adducts, a number of open-source solutions has been developed, e.g.,: DFBuilder, wSIM-City, nLossFinder and FeatureHunter.^15–19^ While their main application is not untargeted LC-MS peak detection, FeatureHunter does allow detection of “pair–peaks” with a fixed mass difference in MS^1^, which is used for identification and profiling using the stable isotope labeling mass spectrometry (SILMS) technique.^18,20^

A software package for untargeted preprocessing of LC-MS data with widespread use is the xmcs R package. It performs untargeted preprocessing of LC-MS data in three steps: (1) peak detection, (2) retention time (RT) alignment and (3) feature grouping.^21^ While this software package is powerful and flexible, the need for data set-specific parameter optimization, of which the importance and impact on research outcome has been described in several studies,^22–24^ remains a hurdle. Furthermore, the processing steps that follow untargeted preprocessing (e.g., normalization, signal drift correction and other data transformations) remain vastly understudied in DNA adductomics.^10,14^ Previous research in other fields emphasizes the importance of proper data processing to remove unwanted variation and obtain high-quality data that can be biologically interpreted.^25,26^ Particularly in LC-MS analyses, where signal drift and batch effects are common and often feature dependent,^27,28^ fit-for-purpose normalization strategies are of paramount importance.

In previous work, we assessed the use of sample vs. feature dependent normalization of DNA adductomics data and illustrated that quality control (QC) normalization was best suited for the management of undesired nonbiological variability in rat tissues compared to total ion count (TIC), median (MedI), internal quality control (iQC) and quality control–based robust LOESS (locally estimated scatterplot smoothing) signal correction (QC-RLSC) normalization.^10^ While providing useful insights, sample size was limited compared to the numbers expected in exposomics studies. Furthermore, both peak detection and evaluation practices were not optimized for the intricacies of DNA adductomics. Hence, in this work, we (re)­evaluated the effectiveness of normalization strategies on more complex, larger-scale analytical batches and optimized xcms parameters for untargeted DNA adductomics data preprocessing. To that purpose, we analyzed the DNA adductome in a subset of placenta samples of the Flemish ENVIR*ON*AGE^29^ birth cohort (*n* = 375) and in a subset of blood samples of the ESCCAPE^30^ study (*n* = 51).

## Material and Methods

### Biological Samples

Placental tissue samples of 375 mother-newborn pairs, part of the ENVIRonmental influence ON early Aging (ENVIR*ON*AGE) birth cohort study, were selected for this work, as well as 51 blood (leukocyte) samples from the Esophageal Squamous Cell Carcinoma African Prevention Research (ESCCAPE) study. More information on both studies and sample collection can be found on pages S1 and S2 of the Supporting Information.

### Chemicals and Reagents

DNA adduct standards M_1_-G (pyrimido­[1,2-*a*]­purin-10­(^1^H)-one), 8-oxo-dG (8-Oxo-2′-deoxyguanosine), Cro-dG (α-methyl-γ-hydroxy-1,N^2^-propano-2′-deoxyguanosine), [^13^C_3_]-M_1_-G, [^13^C,^15^N_2_]-Cro-dG, N^2^-ethyl-dG, (N^2^-ethyl-2′deoxyguanosine), N^6^-Me-A (N^6^-methyl-adenine), and N^3^-Me-A (N^3^-methyl-adenine) were obtained from Toronto Research Chemicals (Toronto, Canada) while N^7^-Me-G (N^7^-methyl-guanine), O^6^-Me-dG (O^6^-methyl-2′-deoxyguanosine), and O^6^-[d3]-Me-dG were purchased from Sigma-Aldrich (St. Louis, MO, USA). O^6^–CM-dG (O^6^-carboxymethyl-2′-deoxyguanosine) was provided by Prof. S. Moore (Liverpool John Moores University (UK)). O^6^–CM-G (O^6^-carboxymethyl-guanine), O^6^-Me-G (O^6^-methyl-guanine), O^6^-[d3]-Me-G, N^2^-ethyl-G (N^2^-ethyl-guanine), Cro-G (α-methyl-γ-hydroxy-1,N^2^-propano-guanine), [^13^C,^15^N_2_]-Cro-G and 8-oxo-G (8-oxoguanine) were obtained by thermal acidic hydrolysis (0.1 M formic acid, 80 °C, 30 min) of their corresponding nucleosides O^6^–CM-dG, O^6^-Me-dG, O^6^-[d3]-Me-dG, N^2^-ethyl-dG, Cro-dG, [^13^C,^15^N_2_]-Cro-dG and 8-oxo-dG. All analytical standards were diluted in MeOH and stored (−20 °C) in stock and working solutions of respectively 500 ng μL^–1^ and 5 ng μL^–1^. Lyophilized Calf Thymus DNA (CT-DNA) was purchased from Rockland (Gilbertsville, Pennsylvania, USA), dissolved in Tris-EDTA buffer and stored at 4 °C (1 mg mL^–1^).

### DNA Adduct Extraction

DNA concentration and purity were measured with an Implen N-60 NanoPhotometer (Implen, Münich, Germany). DNA adduct extraction and analysis were performed as described by Hemeryck et al. (2015).^7^ Following addition of internal standards (ISTDs) O^6^-[d3]-Me-G, [^13^C_3_]-M_1_-G and [^13^C,^15^N_2_]-Cro-G, samples were hydrolyzed in 0.1 M formic acid at 80 °C for 30 min. Next, after cooling down on ice, solid-phase extraction (Oasis HLB cartridges (1 cc, 30 mg), Waters, Milford, CT, USA) was performed. All eluates were dried by evaporation under vacuum at room temperature and resuspended in 100 μL 0.05% acetic acid in H_2_O.

### UHPLC-HRMS Analysis

LC-MS analysis was performed using a hybrid Quadrupole-Orbitrap High Resolution Accurate Mass Spectrometer (HRAM, Q-Exactive, Thermo Fisher Scientific, San José, USA) coupled to a heated electrospray ionization (HESI-II) source as previously described (Hemeryck et al.^7^). A DNA adduct standard mixture was analyzed to check LC and MS performance at the beginning of the MS run sequence. Sample injection volume was 10 μL. QC samples were composed by pooling 10 μL aliquots from each sample. Four external QC samples (eQCs) were analyzed prior to analyzing the samples in randomized order. In between each 10 samples, 2 internal QC samples (iQCs) were analyzed. iQC vial two was considered a technical replicate for evaluation purposes. Following analysis of all samples, 4 eQCs and the DNA adduct standard mixture were analyzed again. Untargeted DNA adduct analysis was enabled by full-scan MS acquisition at 100,000 Full Width Half Maximum in a range of 70 to 700 Da. During the analysis of the ENVIR*ON*AGE samples, instrumental problems required the analyst to switch columns twice midway through the analysis, resulting in three “batches”. The injection sequence of the ENVIR*ON*AGE batches is illustrated in Figure S1 for clarity.

### Optimization of Untargeted Preprocessing Parameters

All data was preprocessed using R (v. 4.4.1) and the xcms R package^21^ (v. 4.3.4). Raw chromatographic data quality of 13 target DNA adducts was inspected in all runs (51 samples and 12 QCs) of the ESCCAPE analysis and respectively 80 and 20 randomly selected sample and QC runs of the ENVIR*ON*AGE analysis (to minimize plot clutter and maintain interpretability). Two endogenous DNA adducts (N^7^-methyl-guanine and 8-oxoguanine), and three internal standard (ISTD) DNA adducts ([^13^C_3_]-M_1_-G, [^13^C,^15^N_2_]-Cro-dG and O^6^-[d3]-Me-dG) showed consistent peak signals in the raw data (based upon visual inspection of the raw data) across both data sets. To ensure reliable peak detection of these compounds, referred to as targets in the remainder of the work, parameters for untargeted detection were optimized in both data sets.

First, parameters for the centWave^31^ algorithm were optimized to achieve successful untargeted peak detection of the five selected targets. Initial assessment of parameters was performed by running the centWave algorithm on the extracted ion chromatograms (EICs) of the five targets in 10 random sample runs. The EICs were extracted using an interval of ±5 ppm and ±15 s around the target’s expected mass-to-charge ratio (*m*/*z*) and RT, respectively. CentWave parameters were optimized through visual assessment of the peak detection results using different sets of parameters. The peakwidth parameter was optimized through visually evaluating the peak width of targets in EICs and choosing the minimum and maximum observed peak widths as parameter values. To optimize the *ppm* parameter, maintainers of xcms recommend to generate a restricted MS window with a single mass peak per spectrum.^32^ The *m*/*z* of target peaks in this area was extracted, their absolute difference calculated and finally expressed in ppm. Other parameters (*integrate, snthresh, extendLengthMSW* and *firstBaselineCheck)* were empirically optimized through trial-and-error until robust detection and integration of the five targets was achieved in their EICs. Parameters *integrate, extendLengthMSW* and *firstBaselineCheck* are binary choices (e.g., TRUE or FALSE); all combinations were evaluated until successful detection was achieved. *snthresh* was incrementally lowered by 1 from its default (10) until successful detection was achieved. Afterward, the centWave algorithm was run in the complete RT and *m*/*z* dimension of the 10 runs instead of in each EIC separately to evaluate if the peaks would still be detected.

Second, parameters for retention time alignment were optimized using the results of centWave peak detection in the aforementioned 10 sample runs. For the ENVIR*ON*AGE data set, the OBI-Warp algorithm^33^ was employed. Performance of the retention time alignment of the 10 samples was judged based on the plots of the EICs of the five targets. The parameter *binSize* was adjusted to a smaller value (0.01 instead of the default 1.00), as we are employing a high-resolution MS.^32^ The parameter *centerSample* was set to “1” to achieve proper alignment. In the ESCCAPE data set, however, employing OBI-Warp worsened alignment, so the PeakGroups^21^ method was employed. In this case, the three ISTD targets were employed as anchor peaks for the alignment. No further parameter optimization was necessary to achieve good alignment in this study.

Lastly, parameters for feature grouping using the PeakDensity^21^ method were optimized using the results after peak detection and retention time alignment in the 10 sample runs. Parameters were optimized to ensure that the five target peaks were grouped into five separate features. Grouping depends on the distribution of peaks from all samples along the RT axis. When peaks with similar RT result in a greater density at a certain RT they are grouped together.^34^ Parameters *bw*, which determines the smoothness of the density curve, and *minFraction*, which defines the minimum proportion of samples within a sample group, were optimized using trial-and-error. Parameters *binSize* and *ppm* were adjusted for use with a high-resolution MS.^32^

### Untargeted Preprocessing

After optimization of parameters, untargeted preprocessing using xcms (peak detection, retention time alignment, feature grouping, and gap filling) was performed. The optimized parameters used to preprocess the complete ENVIR*ON*AGE and ESCCAPE analyses are shown in Table S1. Lastly, the fillChromPeaks­() function (using default parameters) was used to integrate signals from the original data files for samples in which no chromatographic peak was found from the *m*/*z* - RT region where signal from the ion is expected.^21^

### Data Processing and Normalization

In both data sets, features with a missing value in more than 50% of the sample runs were removed. Remaining missing values were imputed by sampling from a uniform distribution that ranges from 1/2 of the smallest measured value to the smallest measured value for the feature.^32^ Principal component analysis (PCA) was performed on log2 transformed, centered and scaled data. In the ENVIR*ON*AGE analysis, the number of sample and QC runs with the second column was too low to enable all normalization methods for all samples; as such, based on PCA score plot clustering (see results and discussion), samples ran with the first and second columns were considered one batch, and samples ran with the third column were considered a separate batch.

Sample based signal correction was performed using total ion count (TIC) normalization and median normalization using the normalizeIntensity­() function implemented in the R package qmtools.^35^ Feature based signal correction using iQC normalization (FBSC-B) and local mean based signal correction (lomec) were performed as described by Kamleh et al.^36^ Linear model based signal adjustment (LMBSC) was performed using the fit_lm­() and adjust_lm­() functions from the MetaboCoreUtils^37^ R package, as described by Wehrens et al.^38^ QC–based robust locally estimated scatterplot (LOESS) smoothing signal correction (QC-RLSC) was performed as described by Dunn et al.,^27^ except the LOESS curve was fitted using the loess.as function from the fANCOVA R package,^39^ which employs generalized cross-validation (GCV) instead of leave-one-out cross-validation (LOOCV). For all methods, normalization was performed by dividing the original values through the normalization factor. To avoid misinterpretation of relative standard deviations (RSD), scaled values were reverted to their original scales, by multiplication with the median of the normalization factors.

For ENVIR*ON*AGE specifically, first within-batch normalization as described above was performed, and second, both batches were aligned by mean response as described by Broadhurst et al.^28^

### Evaluation of Normalization Performance

A robust estimate of relative standard deviations (defined by Broadhurst et al.,^28^ hereafter indicated RSD*) of the peak areas was calculated for each of the three ISTD targets in all sample runs using the rsd­() function (with parameter *mad* = TRUE) from the MetaboCoreUtils^37^ package. RSD*s of the ISTDs for each normalization method were compared to each other as well as to the RSD*s of non-normalized imputed peak areas using the Durbin-Conover pairwise comparison test.^40^ *P*-values were adjusted for multiple comparisons using the Holm method.^41^ *P*-values less than 0.05 were considered to indicate statistical significance.

Thresholds for RSD* and D-ratio were selected based on accepted standards for metabolomics LC-MS experiments^28^ and were more relaxed for ENVIR*ON*AGE due to higher expected variability (larger analysis and instrumental issues during runs). For each normalization method, the numbers of untargeted features with an RSD* in technical replicates lower than 0.2 and lower than 0.3 were counted in the ESCCAPE and ENVIR*ON*AGE data sets, respectively. Differences in RSD* of such features between normalization methods were compared using Dunn’s nonparametric all-pairs comparison test for Kruskal-type ranked data^42^ and the Holm^41^ method to adjust *P*-values for multiple comparisons. D-ratio^28^ (nonparametric alternative, defined in Broadhurst et al.^28^) was calculated for each normalization method based on sample and technical replicate runs using the rowDratio­() function (with parameter *mad* = TRUE) from the MetaboCoreUtils^37^ package. For each normalization method, the numbers of untargeted features with a D-ratio lower than 0.4 and lower than 0.5 were counted in the ESCCAPE and ENVIR*ON*AGE data sets, respectively. Differences in D-ratio between normalization methods were compared using Dunn’s nonparametric all-pairs comparison test for Kruskal-type ranked data^42^ and the Holm^41^ method to adjust P-values for multiple comparisons.

Features with both an RSD* < 0.2 and D-ratio <0.4 in the ESCCAPE data set and an RSD* < 0.3 and D-ratio <0.5 in the ENVIR*ON*AGE data set were retained. In samples, DNA concentration correction was applied as described in De Graeve et al.:^10^ areas were divided by the DNA concentration (ng/μL) and multiplied by the average DNA concentration. Finally, PCA of the different methods was employed also, to visually evaluate QC clustering.

## Results and Discussion

### Optimized xcms Preprocessing for Untargeted DNA Adductomics

The *peakwidth* parameter for centWave was set to a minimum of 4 and 1, and a maximum of 30 and 25 for ENVIR*ON*AGE (placenta) and ESCCAPE (blood), respectively. When evaluating the maximum ppm deviation of the target peaks, differences were observed between the two data sets. For example, the maximum deviation for 8-oxoguanine was 0.91 ppm in the evaluated ENVIR*ON*AGE runs and 4.8 ppm in the ESCCAPE runs. Results indicate that the instrument is capable of sufficiently accurate measurements of DNA adducts, as deviation for none of our targets exceeded 5 ppm (in the evaluated runs). As such, to disregard lower accuracy measurements, the vendor-advertised value of 5 ppm was chosen for both data sets. Settings for all other centWave parameters that resulted in successful detection and integration of the targets (examples in Figure 1) in both data sets can be found in Table S1.

**Figure 1:**
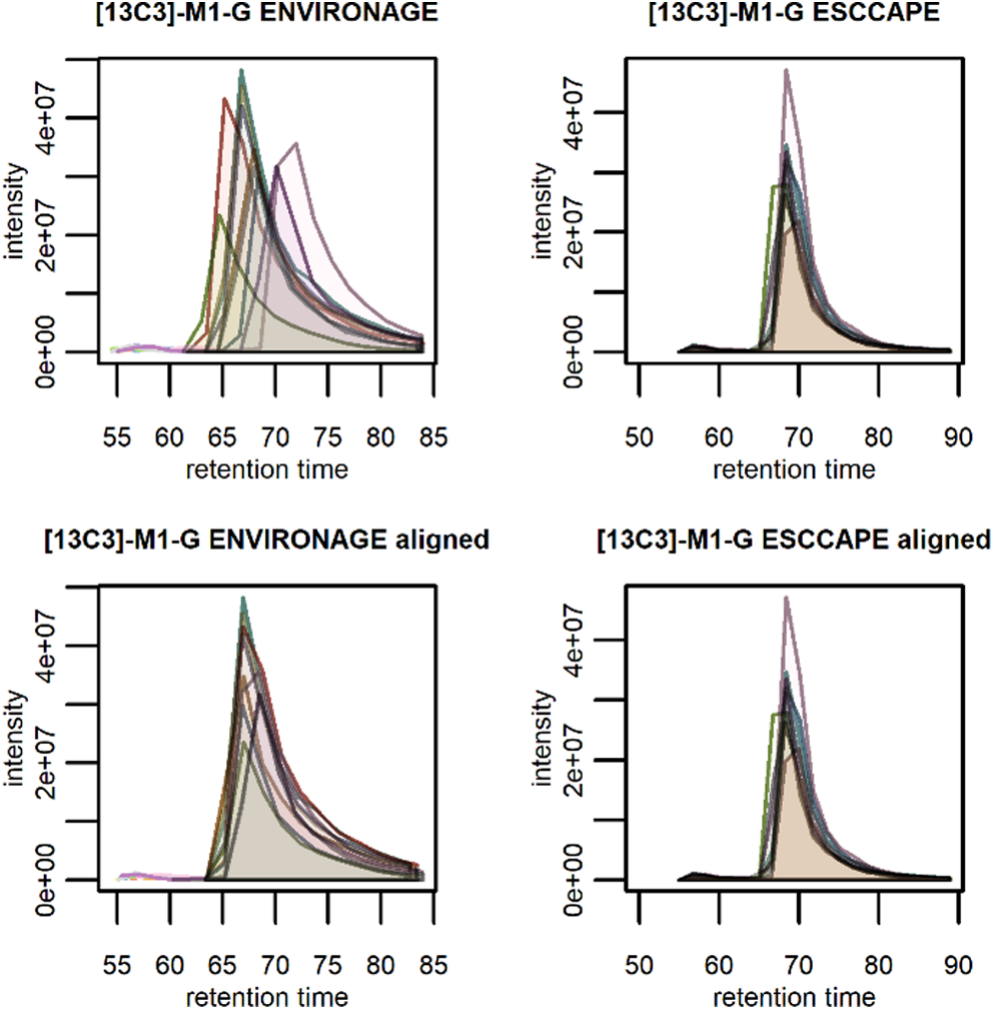
Illustration of results of RT alignment for well-integrated peaks of [^13^C_3_]-M_1_-G in 10 sample runs in both the ENVIRONAGE and ESCCAPE data set. RT is shown in seconds, intensity in arbitrary units. The upper two panels show the EICs in unadjusted data, the bottom two panels show the EICs after alignment.

For the ENVIR*ON*AGE data set, even in the subset of only 10 samples, RT shifts of the target peaks were visible (Figure 1). Particularly, the EICs of [^13^C,^15^N_2_]-Cro-dG illustrated the need for RT alignment to achieve proper feature grouping (Figure S2). Many RT alignment algorithms for LC-MS data are available, of which some have been implemented in xcms.^43^ For the ENVIR*ON*AGE data set, the OBI-warp algorithm was selected, because it supports alignment of multiple samples against a center sample and may be performed independently of peak detection and grouping.^33^ Good alignment was achieved, by defining the center sample through trial-and-error (parameter *centerSample)* (Figure S2 shows result of alignment with a nonsuited center sample). Users of the OBI-warp method are thus encouraged to carefully select the center sample for their data set and visually evaluate RT alignment for important targets. Inspecting the EICs of the targets in the ESCCAPE data set, little to no RT shifts could be observed. Moreover, when applying the OBI-warp algorithm in the ESCCAPE data set, alignment worsened, even after testing every run as center sample (Figure S3). Therefore, the peakGroups^21^ method was applied using the ISTD targets as anchor peaks. The mean absolute difference between the adjusted and raw retention times was 4.28 s for ENVIR*ON*AGE and 0 s for ESCCAPE, indicating that the ESCCAPE analysis did not benefit from further alignment compared to using its raw retention times.

Feature grouping was achieved through the PeakDensity^21^ approach, which clusters chromatographic peaks with similar *m*/*z* and RT values into discrete features. For both data sets, a *bw* value of 2 resulted in correct grouping of the target peaks, while minimizing the risk of falsely grouping peaks into a feature. The effect of *bw* is illustrated in Figures S4–S6. Another important parameter that determines the number of features reported is *minFraction*, which defines the minimum proportion of runs within a sample group in which peaks need to be detected in order to be grouped as a feature. In these analyses, sample and QC runs are distinguished as two “sample” groups. In DNA adductomics, it is advisible to set this parameter to a low value, since it is expected that certain DNA adducts of interest will be present in a minority of samples, while in QC runs, detection rates are expected to be lower because of dilution effects.^28,44^ In this study, *minFraction* parameters of 0.1 and 0.05 were chosen, so that all targets were grouped into features for the ENVIR*ON*AGE and ESCCAPE analyses, respectively. The value was lower for ESCCAPE to group the peaks N7-methyl-guanine into a feature, as peaks were detected in 7.8% of the ESCCAPE samples. By applying the fillChromPeaks­() function, removal of such peaks due to a large percentage of missing values is avoided.

The results presented above highlight the need for careful optimization of algorithm parameters in xcms. Valuable tools for automatic optimization^23,45^ exist (e.g., IPO), although they have not been evaluated in DNA adductomics studies yet, and should be used with care in data sets with poor chromatographic performance.^24^ Furthermore, some considerations, such as choosing a low *minFraction* parameter, differ substantially from what one would typically choose in e.g., metabolomics experiments (e.g., 0.5 in sample runs and 1 in QC-runs).^24,46^ It is highly recommended for researchers to optimize their parameters per data set, for which they can be referred to the detailed documentation available on e.g., the Metabonaut Web site,^47^ which also served as the basis for the optimization process in this paper. Notably, the results of OBI-warp alignment in the ESCCAPE data set illustrate that the choice of method and parameters can adversely lead to a lower quality data set for downstream processing, further stressing the need for data set-specific optimization of preprocessing parameters.

### Untargeted DNA Adductome Peak Picking

For ENVIR*ON*AGE, untargeted preprocessing generated more than 7.5 million chromatographic peaks detected in 457 runs, grouped into 15.781 features. In the ESCCAPE data set, 1.1 million peaks were detected in 65 runs and grouped into 20.015 features. The substantially higher number of features in ESCCAPE can be attributed to the lower setting of the *minFraction* parameter discussed earlier. A total of 617 and 2846 features had more than 50% missing values (after use of the fillChromPeaks­() function) in samples in ENVIR*ON*AGE and ESCCAPE respectively and were removed. All five targets for which the parameters were optimized could be retrieved in the feature list of both data sets after filtering. As expected, and confirming the robustness of the optimized preprocessing approach, ISTD target signal was retrieved in all but two runs (Table S2).

### Handling Batch Effects in the Untargeted DNA Adductome

Exploratory PCA revealed a clear batch effect in the ENVIR*ON*AGE analysis, corresponding to the three column changes during the analysis and the hence induced chromatographic shifts (see Material and Methods and Figure 2). Applying alignment by mean response after within-batch normalization (illustrated in Figure 2 for QC-RLSC), as described by Broadhurst et al.,^28^ removed the batch effect successfully. Inspecting score plots including PC2 and PC3 did show some residual batch effect; samples analyzed with column 1 and 3 appeared to be more similar, yet clear clustering as in Figure 2 was absent (Figures S7 and S8). In the ESCCAPE analysis, the observed dependency on injection order was successfully removed after normalization (Figure S9) and therefore, alignment by mean response was not applied.

**Figure 2:**
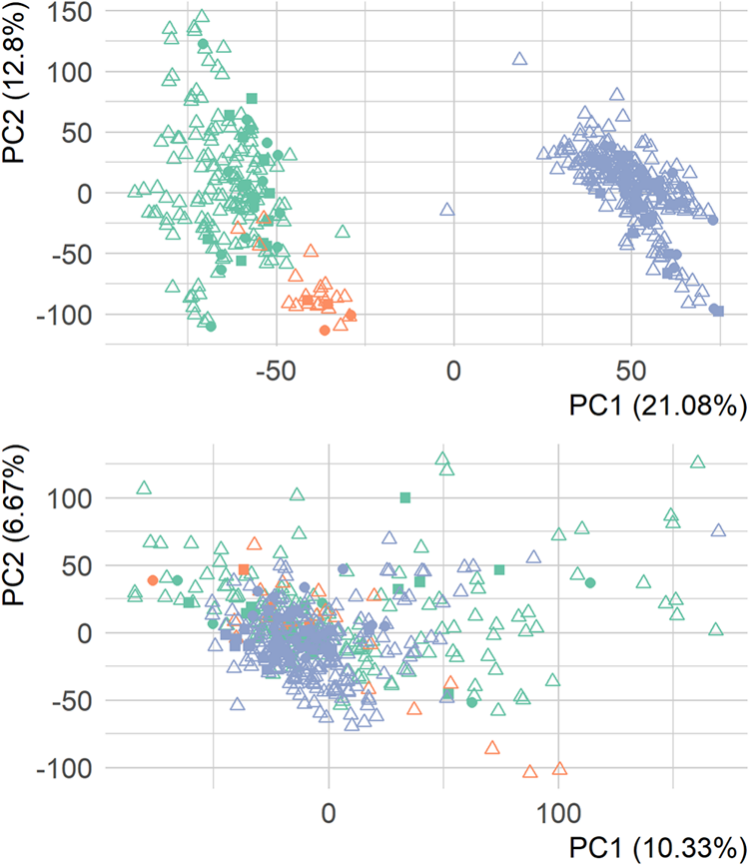
Upper panel shows the PCA score plot of non-normalized measured areas of untargeted features in the ENVIR*ON*AGE placenta sample analysis. The bottom panel shows the PCA for the same data after QC-RLSC normalization and batch effect correction. Colors correspond to the three columns (green: 1, orange: 2, and blue: 3). Shape indicates run type (circle: QC, triangle: sample, square: technical replicate).

### Evaluation of Normalization Methods to Remove Unwanted Variation in Untargeted DNA Adductomics

In previous work, De Graeve et al. evaluated the performance of normalization strategies in DNA adductomics by evaluating QC sample clustering in the PCA and features’ standard deviations in samples.^10^ Both approaches were inspired by good practices in

metabolomics,^10,26,48^ but, since it is expected that DNA adducts are often only present in a small percentage of samples in a (healthy) cohort,^49^ the standard deviation of features in samples is expected to be high, without implying poor analytical quality. While lower standard deviations should be expected after normalization, favoring the method that reduces standard deviations in samples (i.e., the combination of technical and biological variation) the most, does not discern between reduction in technical or biological variation. In addition, evaluation of QC runs could lead to a bias that favors methods employing QCs for calculating correction factors.^36^ To overcome this bias, Kamleh et al.^36^ proposed the use of technical replicates or QC samples other than the ones being used for the calculation of normalization factors. Therefore, in this work it was chosen to evaluate normalization efficiency using RSD* and D-ratio of ISTDs in samples and of untargeted features in QCs not employed for normalization. This is an important consideration, and while already described in 2012 by Kamleh et al.,^36^ it is seldom applied in recent DNA adductomics and metabolomics (normalization) studies alike.^10,50–52^

Pairwise comparison of the RSD*s of the ISTDs (Figure 3 and Tables S3 and S4) between the different normalization methods revealed a comparable trend in the two data sets. Feature-based signal correction methods (FBSC-B, lomec, LMBSC and QC-RLSC) consistently outperformed the sample-based methods (TIC and median). Sample-based correction methods inflated the RSD* of ISTD targets significantly compared to the non-normalized data, indicating the introduction of unwanted variance in the data. Within the feature-based methods, LMBSC performed the worst. In both data sets, it showed no statistically significant difference compared to non-normalized data. Indeed, LMBSC as implemented in lm_adjust­() only performs correction if a statistically significant linear relation is observed between the feature signal and injection index.^38^ This result indicates that the signal more often than not has a nonlinear relation with injection index. This clarifies the better performance of methods that use more complex fitting procedures such as QC-RLSC, or model the drift more locally, such as FBSC-B and lomec. Of these three methods, QC-RLSC and lomec lowered RSD* statistically significantly more than FBSC-B.

**Figure 3:**
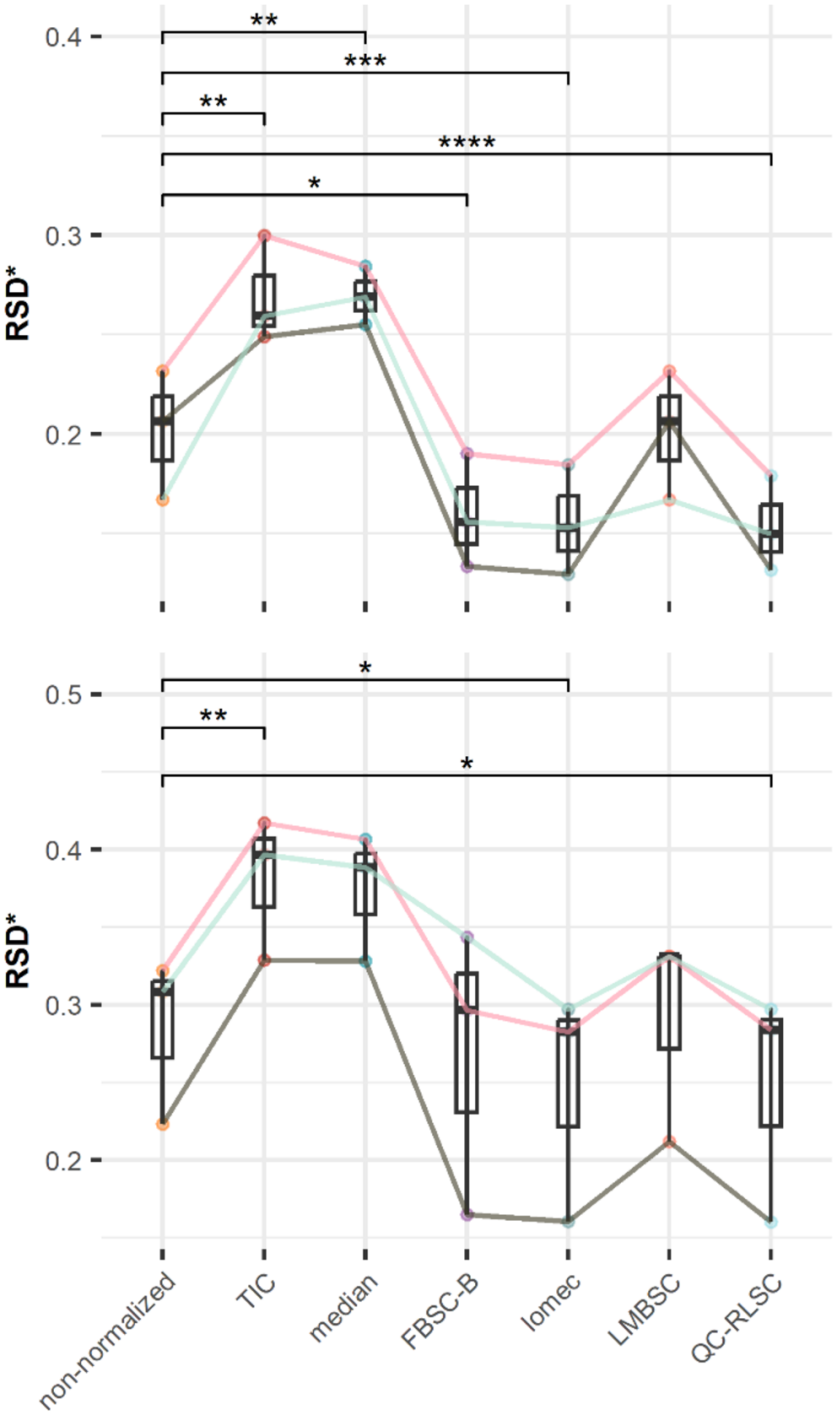
Boxplots of paired pairwise comparisons of the RSD* of three ISTD target peaks in ESCCAPE (upper panel, RSD* calculated in 51 sample runs) and ENVIR*ON*AGE (lower panel, RSD* calculated in 375 sample runs). Significant differences versus the non-normalized data are indicated with asterisks: **** < 1e-04, *** < 0.001, ** < 0.01, * < 0.05.

The number of untargeted features with an RSD* below the accepted thresholds in technical replicates^28^ for each method can be consulted in Tables S5 and S6. For both data sets, employing QC-RLSC led to the highest number of features with an RSD* below the threshold (8329 and 2999 for ESCCAPE and ENVIR*ON*AGE, respectively). In ESCCAPE, median normalization led to the lowest number of features below the threshold (4818), while interestingly, in ENVIR*ON*AGE, FBSC-B led to the lowest number (1973). Comparisons between RSD*s of features that were below the threshold after different normalization methods can be found in Figures S10 and S11, and significant differences (*p* < 0.05) are listed in Tables S7 and S8. In ESCCAPE, feature-based methods not only led to a higher number of features below the threshold but also led to retained features having a lower RSD* compared to the two sample-based methods. This result could not be replicated in ENVIR*ON*AGE, where the RSD* of features differed significantly in only few methods (Table S8). Feature-based methods, however, visually showed more outliers toward features with low RSD*s (Figure S11).

The number of untargeted features with a D-ratio below the threshold (0.4 for ESCCAPE and 0.5 for ENVIR*ON*AGE) for each method can be found in Tables S9 and S10. Results for ESCCAPE are in line with previous findings, as feature-based methods led to the highest number of features below the threshold. In ENVIR*ON*AGE however, TIC normalization led to the highest number of features below the threshold (1220) and also median normalization led to more retained features than lomec, LMBSC and QC-RLSC. Inspecting the formula for D-ratio, as defined by Broadhurst et al.^28^

1 $${D‐\mathrm{ratio}}_{i}=\frac{{\mathrm{MAD}}_{i,\mathrm{rep}}}{{\mathrm{MAD}}_{i,\mathrm{sample}}}$$

we hypothesize that sample-based methods inflate the biological variation of features more than it does the technical variation in our data set, hence leading to a higher mean absolute deviation (MAD) in samples and a lower D-ratio. This hypothesis was validated as we observed a larger increase of RSD* of ISTD compounds in samples compared to in technical replicates (Figure S12). Other studies also reported on limitations of sample-based methods such as TIC and median normalization, especially when there is no roughly equal number of features being up- or downregulated.^53,54^ Based on these results, care should be taken when employing sample-based normalization methods in untargeted DNA adductomics and other untargeted LC-MS applications. Especially if features are afterward filtered based on D-ratio, downstream statistics could lead to spurious results because of the artificial inflation of variance in biological samples.

The PC score plots (Figures S13 and S14) for the retained features per normalization method however illustrate that quantitative metrics do not provide the full picture. Indeed, in the PCA score plot for TIC normalized data of ENVIR*ON*AGE (Figure S14), both QC and technical replicates do not cluster tightly in comparison to the total variance. This is contrary to what would be expected of a high-quality data set.^28^ Also, previous results showed that TIC artificially inflated variation of the ISTD targets (Figure 3). Comparisons between D-ratio of features that were below the threshold following different normalization methods are presented in Figures S15 and S16, and significant differences (*p* < 0.05) are listed in Table S11.

The total number of features retained following each normalization method in both data sets, meeting both the thresholds for RSD* and D-ratio, are listed in Tables S12 and S13. Based on subjective evaluation of PCA score plots and number of features meeting the RSD* and D-ratio thresholds, QC-RLSC was the selected normalization method for the ENVIR*ON*AGE data set. While indeed QC-RLSC did not retain the highest number of features, data quality was deemed significantly better based on the PCA score plots (Figure S14). Interestingly, for ESCCAPE, results were more nuanced. The score plot for lomec normalized features showed good clustering for both QCs and replicates, although one QC was separated in the PC2 dimension (Figure S13). Clustering was better for QC-RLSC normalized data, for which 2736 features were retained (compared to 2819 in lomec). Here, for demonstration purposes, lomec was finally chosen, but the choice is debatable.

A DNA adductome map of all retained features using the selected normalization method is presented in Figure S17 for both data sets. Evaluation of the resulting features in both data sets revealed that two of the ISTDs employed to optimize preprocessing were retained after RSD* and D-ratio filtering: [^13^C,^15^N_2_]-Cro-dG and [^13^C_3_]-M_1_-G in the ESCCAPE and ENVIRONAGE data set, respectively. Since for ISTDs, the variance between a technical replicate and sample run is not expected to differ, the results were evaluated again without D-ratio filtering. All ISTDs were retained in both data sets with an RSD* < 0.3, except [^13^C_3_]-M_1_-G (RSD* > 0.3 and < 0.4) in the ESCCAPE cohort. Also disregarding D-ratio, both endogenous compounds were retrieved with an RSD* < 0.3 in the ESCCAPE data set, but not in the ENVIR*ON*AGE data set. Since the endogenous compounds were lost through filtering, it could be argued that the thresholds are too stringent for our data sets, However, for untargeted DNA adductomics in the exposomics context, reliably detected features (RSD* < 0.3) with sufficient variation in samples (D-ratio <0.5) are still favored for further analysis compared to known, but less reliably measured endogenous compounds. Additionally, further relaxation of the thresholds to include said endogenous compounds would come at the cost of an increase of noisy features, complicating further analysis and/or annotation.

### Proposed Workflow for Untargeted DNA-Adductomics in Exposome Research

Combining all previously described results, our proposed and successfully applied workflow is presented in Figure 4.

**Figure 4:**
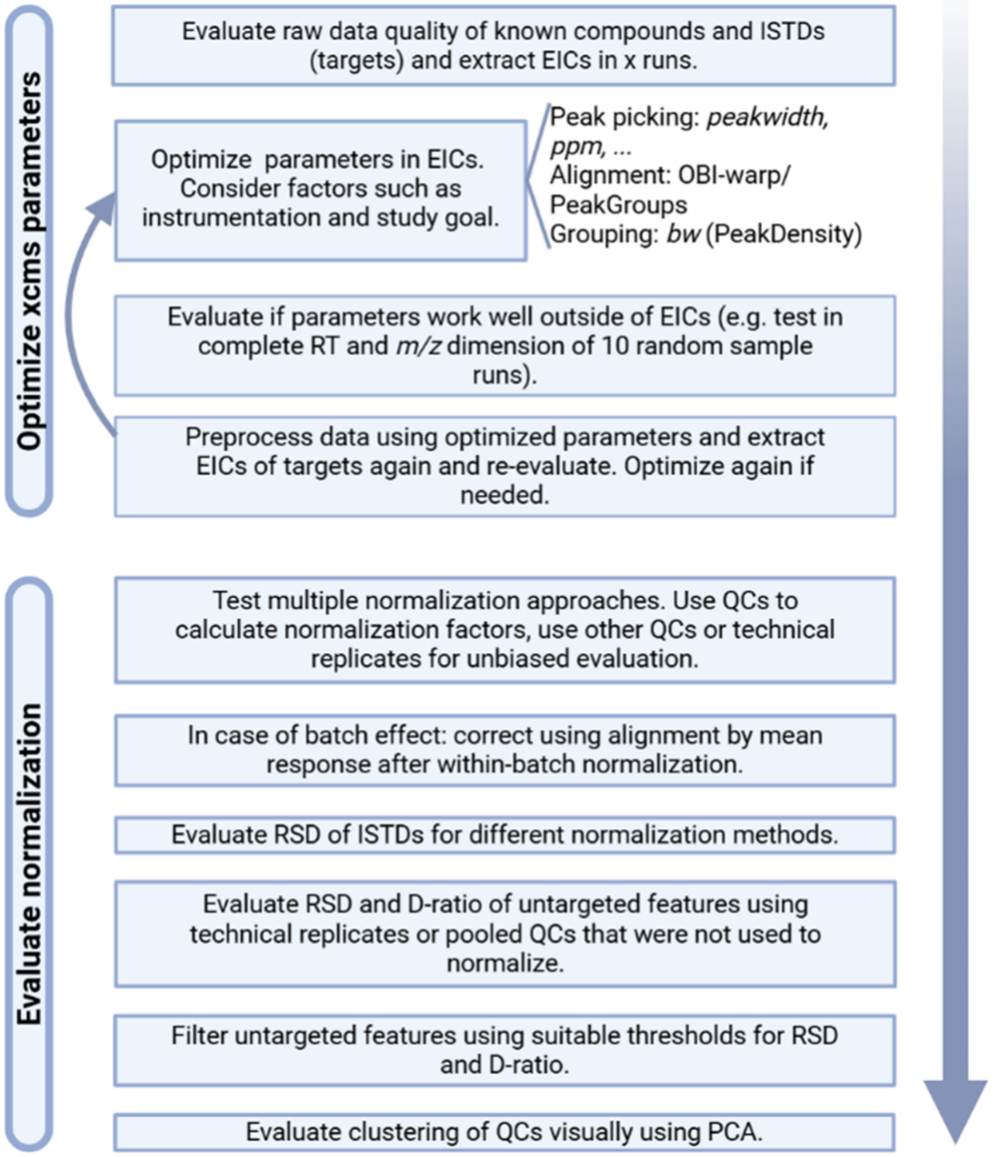
Proposed workflow for data preprocessing of LC-MS DNA adductomics data in exposome research.

### Strengths and Limitations

This is the first study to report implementation of DNA adductomics in a large-scale exposomics setting, with analysis of more than 350 samples in a single sample series. Although xcms has been applied for untargeted DNA adductomics in an LC-MS/MS context,^8^ this work is - to the authors’ knowledge - also the first to provide in-depth description of optimization of parameters in a large-scale LC-MS DNA adductomics context. Leveraging this large amount of biological data, this work provides a reproducible framework for both data preprocessing and evaluation of normalization strategies, which has shown to be robust despite analytical challenges. Furthermore, our results revealed limitations of the D-ratio metric, especially for sample-based normalization methods.

The authors acknowledge that the need for relaxation of thresholds in the ENVIRONAGE data set stems from variation due to unforeseen technical issues during the analysis (i.e., the batch effect due to column changes), which could not be remedied. Furthermore, the authors are aware of the limitations of optimizing preprocessing based on five target compounds, as only two DNA adducts could reliably be detected in samples. This is due to the fact that (1) DNA adducts are low-abundant biomolecules compared to e.g., metabolites, in particular in healthy populations,^49,55,56^ and (2) only few analytical standards are available.^57,58^

## Conclusions

This work aimed to provide a fit-for-purpose framework for data preprocessing and normalization of untargeted DNA adductomics data. Optimization of xcms parameters allowed for reliable, untargeted detection of known DNA adducts. The parameters described in the methods and results should not be considered as gold standards for untargeted DNA-adductomics experiments; i.e., researchers are encouraged to find optimal parameters for their data sets and, if possible, use a greater number of ISTDs. Quantitative evaluation of normalization methods demonstrated the importance of data set-specific evaluation and method choice, and revealed important considerations concerning the use of metrics such as RSD* and D-ratio. This study showed that, while important as an objective measure, these metrics also have their shortcomings. As such, qualitative evaluations such as PCA score plots, while subjective, remain a must. With this work, we provide a first step toward transparent and reproducible data preprocessing for untargeted DNA adductomics in exposome research.

## Supplementary Material



## Notes

Cohort information and Supporting Figures and Tables (PDF)
