# Supplementary material for: Challenges and Good Practices in Preprocessing and Normalization of Untargeted DNA Adductomics Data in Exposomics Research
Source: Anal Chem. 2026 Mar 16;98(12):8947–55. doi: 10.1021/acs.analchem.5c06549 (PMC13147328; doi:10.1021/acs.analchem.5c06549)
Supplement: Supplementary file 1 [file ac5c06549_si_001.pdf]

Supporting Information

Challenges and good practices in preprocessing and normalization of untargeted DNA adductomics data in exposomics research

Pablo Vangeenderhuysen[1], Matthijs Vynck[1], Liesa Engelen[2], Adrian Covaci[3], Tim Nawrot[2,4], Trancizeo Lipenga[5,6], Roger Pero-Gascon[5,7], Sarah De Saeger[5,8], Marthe De Boevre[5], Valerie McCormack[9], Lynn Vanhaecke[1,10,*], Lieselot Y. Hemeryck[1]

[1] Laboratory of Integrative Metabolomics (LIMET), Ghent University, 9820 Merelbeke, Belgium

[2] Centre for Environmental Sciences, Hasselt University, 3590 Diepenbeek, Belgium

[3] Toxicological Centre, University of Antwerp, 2610 Wilrijk, Belgium

[4] Department of Public Health & Primary Care, Occupational & Environmental Medicine, KU Leuven, 3000 Leuven, Belgium

[5] Department of Bioanalysis, Centre of Excellence in Mycotoxicology and Public Health, Ghent University, 9000 Ghent, Belgium

[6] Department of Biomedical Sciences, Mzuzu University, P/BAG 201 Luwinga, Mzuzu, Malawi

[7] Department of Chemical Engineering and Analytical Chemistry, Institute for Research on Nutrition and Food Safety (INSA-UB), University of Barcelona, 08007 Barcelona, Spain

[8] Department of Biotechnology and Food Technology, Faculty of Science, University of Johannesburg, Doornfontein Campus, Gauteng, 2094 Johannesburg, South Africa

[9] Environment and Lifestyle Epidemiology Branch, International Agency for Research on Cancer (WHO-IARC), 69007 Lyon, France

[10] Institute for Global Food Security, Queen's University Belfast, BT7 1NN Belfast, United Kingdom

[*] To whom correspondence should be addressed.

Contents

Cohort descriptions S2

Supplementary Figures and Tables S3

Cohort descriptions

ESCCAPE dataset:

Blood samples (n = 300, 150 cases and 150 controls) were collected as part of the Oesophageal Squamous Cell Carcinoma African Prevention Research (ESCCAPE) study. Patients and healthy donors were recruited at the Moi Teaching and Referral Hospital in Eldoret, Kenya, and the Kilimanjaro Clinical Medical Centre in Moshi, Tanzania (including patients from three peripheral hospitals). the ESCCAPE study was coordinated by The International Agency for Research on Cancer (IARC-WHO). It aimed to investigate environmental and lifestyle factors associated with an increased risk of ESCC in the African EC corridor. Cases (≥ 18 years) had histologically confirmed ESCC (90% with endoscopy) or were diagnosed via barium swallow/clinical diagnosis (10%). Controls were recruited in a 1:1 ratio with cases, matching the cases in age and sex. Controls were selected from the same hospitals as the cases. All participants provided signed informed consent. The study received ethical approval from the IARC Ethics Committee (IEC 14/15) and institutional review boards: the Institutional Research and Ethics Committee of Moi University (000921), Kenya; the National Institute for Medical Research (NIMR/HQ/R.8a/Vol.IX/1994) and the Tumaini University Kilimanjaro Christian Medical University College, Tanzania. Ethical approval to perform DNA adductomics analysis was obtained from the Ethics Committee (EC) of Ghent University and Ghent University Hospital (ONZ-2024-0228), Belgium. Of the 300 samples, 51 contained sufficient DNA to be included in the presented work.

ENVIRONAGE dataset:

The ENVIRonmental influence ON early AGEing (ENVIR*ON*AGE) birth cohort study recruited more than 1,600 mother-child pairs in the South-East Limburg Hospital (Genk, Belgium), with follow-up of children during early childhood and preadolescence. Mother-newborn pairs were recruited when they arrived for delivery at the East-Limburg Hospital in Genk (Belgium), following procedures approved by the Ethical Committee of Hasselt University and the East-Limburg Hospital. Biological samples and metadata were collected, including e.g. placental tissue and maternal blood at birth, faecal, urine and blood samples at age 4-6 years, mothers' lifestyle and socio-economic status, clinical data, etc. For the current setup, biobanked placental tissue samples of 375 mother-newborn pairs were selected (MTA- A21-TT-1408). To minimize the impact of within-placental variability, biopsies were all taken 1.5 cm below the chorio-amniotic membrane at a fixed location by using a device to orientate the fetal side of the placenta in relation to the umbilical cord. DNA from up to 100 mg of placental tissue was extracted at the Biobank of UZ Gent (Ghent, Belgium, ID: BE 71067049) by means a Promega Maxwell RSC (Madison, USA), using the Maxwell® RSC Tissue DNA Kit (AS1610).

Supplementary Figures and Tables

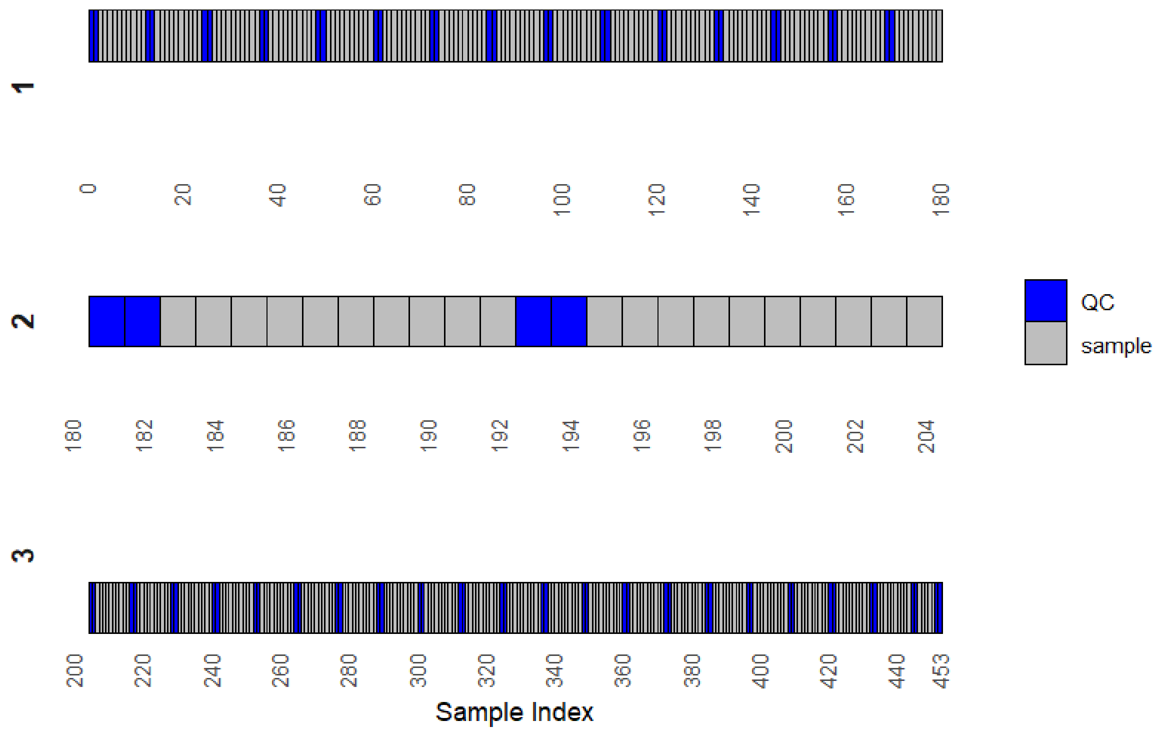

Figure S1. *A schematic overview of the injection and analysis sequence of the ENVIRONAGE samples. Injection index is shown on the x-axis. The three rows indicate the three different columns that were used throughout the analysis.*

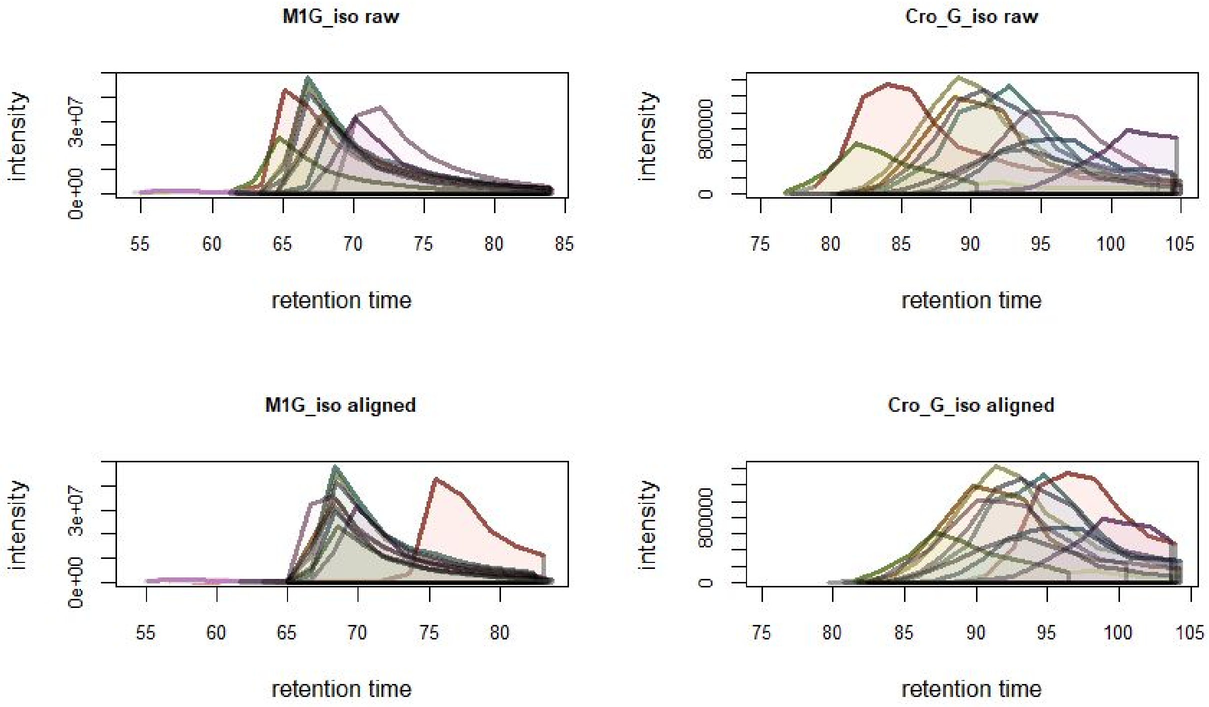

Figure S2. *Retention time alignment results for [$^{13}C_3$]-M_1-G (left) and [^{13}C,$^{15}N_2$]-Cro-G (right) in 10 sample runs of the ENVIRONAGE analysis using a non-suited center sample.*

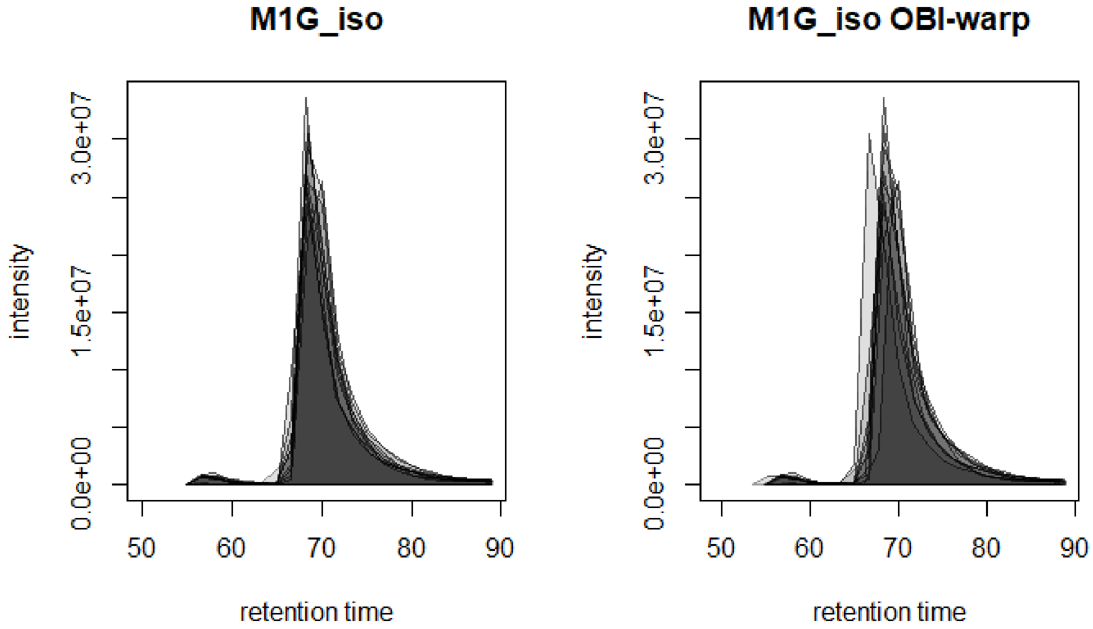

Figure S3*. The effect of OBI-warp alignment of the peak corresponding to [$^{13}C_3$]-M_1-G in the ESCCAPE dataset. The left panel shows the data without any alignment applied. The right panel shows that alignment using OBI-warp leads to misalignment of the peak signal.*

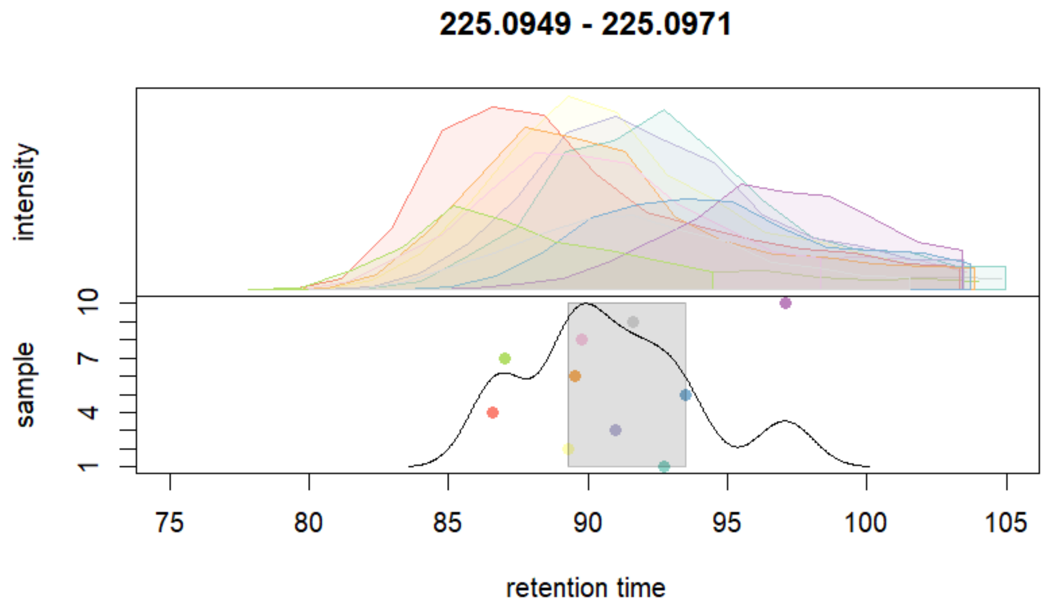

Figure S4. *The EICs of [^{13}C,$^{15}N_2$]-Cro-G in 10 sample runs of the ENVIRONAGE analysis are displayed in the upper panel. In the bottom panel, the estimation distribution (density curve) is displayed. All peaks in samples within a peak of the density curve are grouped into a feature. Here, the bw parameter is chosen too small (1), and thus the density curve is not smoothed enough for all peaks from the 10 samples to be grouped into one feature.*

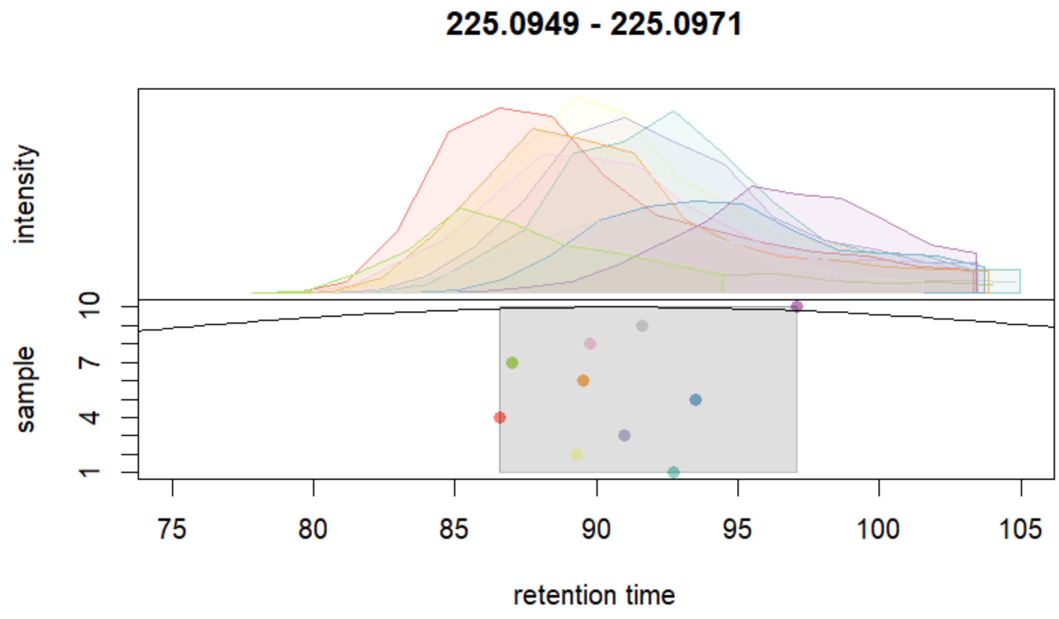

Figure S5. *The EICs of [^{13}C,$^{15}N_2$]-Cro-G in 10 sample runs of the ENVIRONAGE analysis are displayed in the upper panel. In the bottom panel, the estimation distribution (density curve) is displayed. All peaks in samples within a peak of the density curve are grouped into a feature. Here, the bw parameter is chosen too large (30, the default value), and thus the density curve is too smooth, implying that its peak will also include peaks with an RT that is too different from the expected RT.*

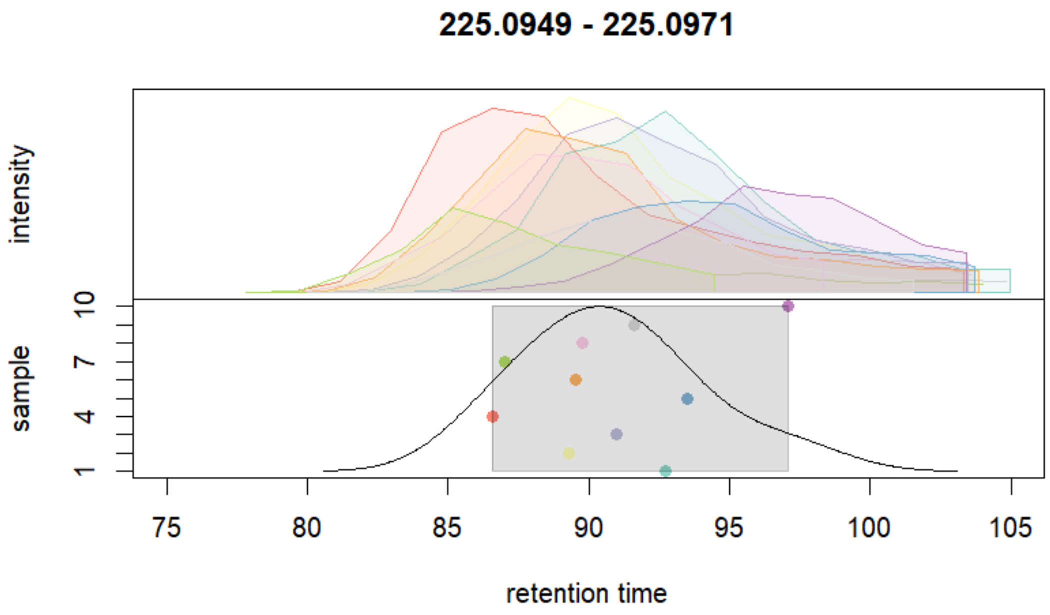

Figure S6. *The EICs of [^{13}C,$^{15}N_2$]-Cro-G in 10 sample runs of the ENVIRONAGE analysis are displayed in the upper panel. In the bottom panel, the estimation distribution (density curve) is displayed. All peaks in samples within a peak of the density curve are grouped into a feature. Here, the bw parameter is chosen appropriately (2), and thus the density curve is smooth enough to include all relevant peaks, while minimizing the risk of including peaks at too different RTs.*

Table S1*. Final set of xcms parameters used to preprocess the complete ENVIRONAGE and ESCCAPE datasets. If a parameter is not listed, the default value was used.*

**Algorithm**	**parameter**	**ENVIRONAGE**	**ESCCAPE**
CentWave	ppm	5	5
CentWave	peakwidth	4, 30	1,25
CentWave	snthresh	3	3
CentWave	prefilter	3, 100	3, 100
CentWave	mzCenterfun	“wMean”	“wMean”
CentWave	integrate	2	2
CentWave	mzdiff	-0.001	-0.001
CentWave	fitgauss	FALSE	FALSE
CentWave	noise	0	0
CentWave	verboseColumns	FALSE	FALSE
CentWave	roiList	List()	List()
CentWave	firstBaselineCheck	FALSE	TRUE
CentWave	roiScales	Numeric(0)	Numeric(0)
CentWave	extendLengthMSW	TRUE	TRUE
CentWave	verboseBetaColumns	FALSE	FALSE

ObiWarp	binSize	0.01	/
ObiWarp	centerSample	1	/
ObiWarp	response	1	/
ObiWarp	distFun	"cor_opt"	/
ObiWarp	gapInit	Numeric(0)	/
ObiWarp	factorDiag	2	/
ObiWarp	factorGap	2	/
ObiWarp	localAlignment	FALSE	/
ObiWarp	initPenalty	0	/
ObiWarp	subset	0	/
ObiWarp	subsetAdjust	"average"	/
ObiWarp	rtimeDifferenceThreshold	5	/
PeakGroups	peakGroupsMatrix	/	Matrix with RT of ISTD compounds
PeakDensity	sampleGroups	"QC" or "sample"	"QC" or "sample"
PeakDensity	bw	2	2
PeakDensity	minFraction	0.1	0.05
PeakDensity	minSamples	1	1
PeakDensity	binSize	0.01	0.01
PeakDensity	maxFeatures	50	50
PeakDensity	ppm	0	0

***Table S2**. Results of untargeted feature detection for the five targets in ENVIRONAGE (upper 5 rows) and ESCCAPE (lower 5 rows).*

**ENVIRONAGE**	**target**	**mzmed**	**mzmin**	**mzmax**	**rtmed**	**rtmin**	**rtmax**	**npeaks**	**# QCs detected**	**# samples detected**
	8-oxo-guanine	168.0513	168.0510	168.0592	56.3	53.8	58.5	170	12	158
	N7-methyl-guanine	166.0722	166.0719	166.0725	61.7	55.0	65.2	59	0	58
	O6-[d3]-Me-dG	169.0909	169.0855	169.0941	70.6	64.8	90.1	678	82	375
	([13C3]-M1-G	191.0626	191.0592	191.0667	68.6	58.3	78.8	963	82	375
	[13C,15N2]-Cro-dG	225.0956	225.0946	225.1013	90.9	68.7	109.2	460	82	373
**ESCCAPE**	**target**	**mzmed**	**mzmin**	**mzmax**	**rtmed**	**rtmin**	**rtmax**	**npeaks**	**QC**	**sample**
	8-oxo-guanine	168.0502	168.0500	168.0504	66.6	64.9	66.8	65	14	51
	N7-methyl-guanine	166.072	166.0719	166.0721	62.4	58.2	63.2	4	0	4
	O6-[d3]-Me-dG	169.0909	169.0908	169.0910	75.1	73.3	76.8	65	14	51
	([13C3]-M1-G	191.0662	191.0661	191.0663	70.0	69.7	71.8	65	14	51
	[13C,15N2]-Cro-dG	225.0953	225.0952	225.0955	100.9	97.6	107.5	65	14	51

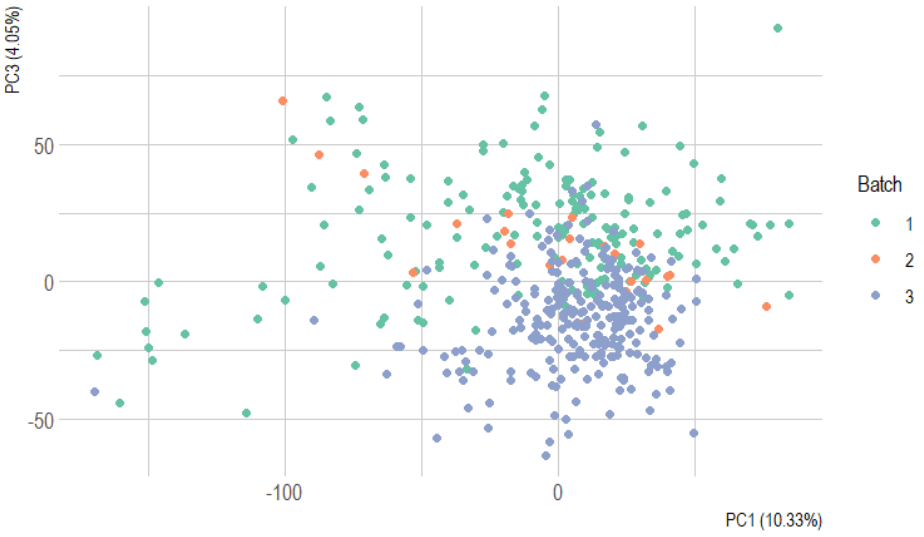

Figure S7. *PCA score plot of PC1 and PC3 of measured areas of untargeted features after QC-RLSC normalization and batch effect correction in the ENVIRONAGE placenta sample analysis. Colors correspond to the three columns (green: 1, orange: 2, and blue: 3).*

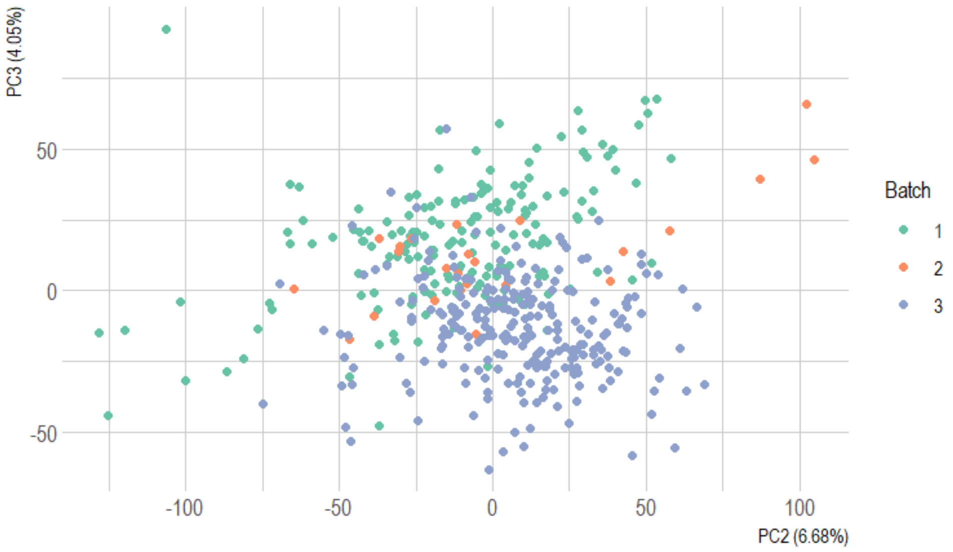

Figure S8. *PCA score plot of PC2 and PC3 of measured areas of untargeted features after QC-RLSC normalization and batch effect correction in the ENVIRONAGE placenta sample analysis. Colors correspond to the three columns (green: 1, orange: 2, and blue: 3).*

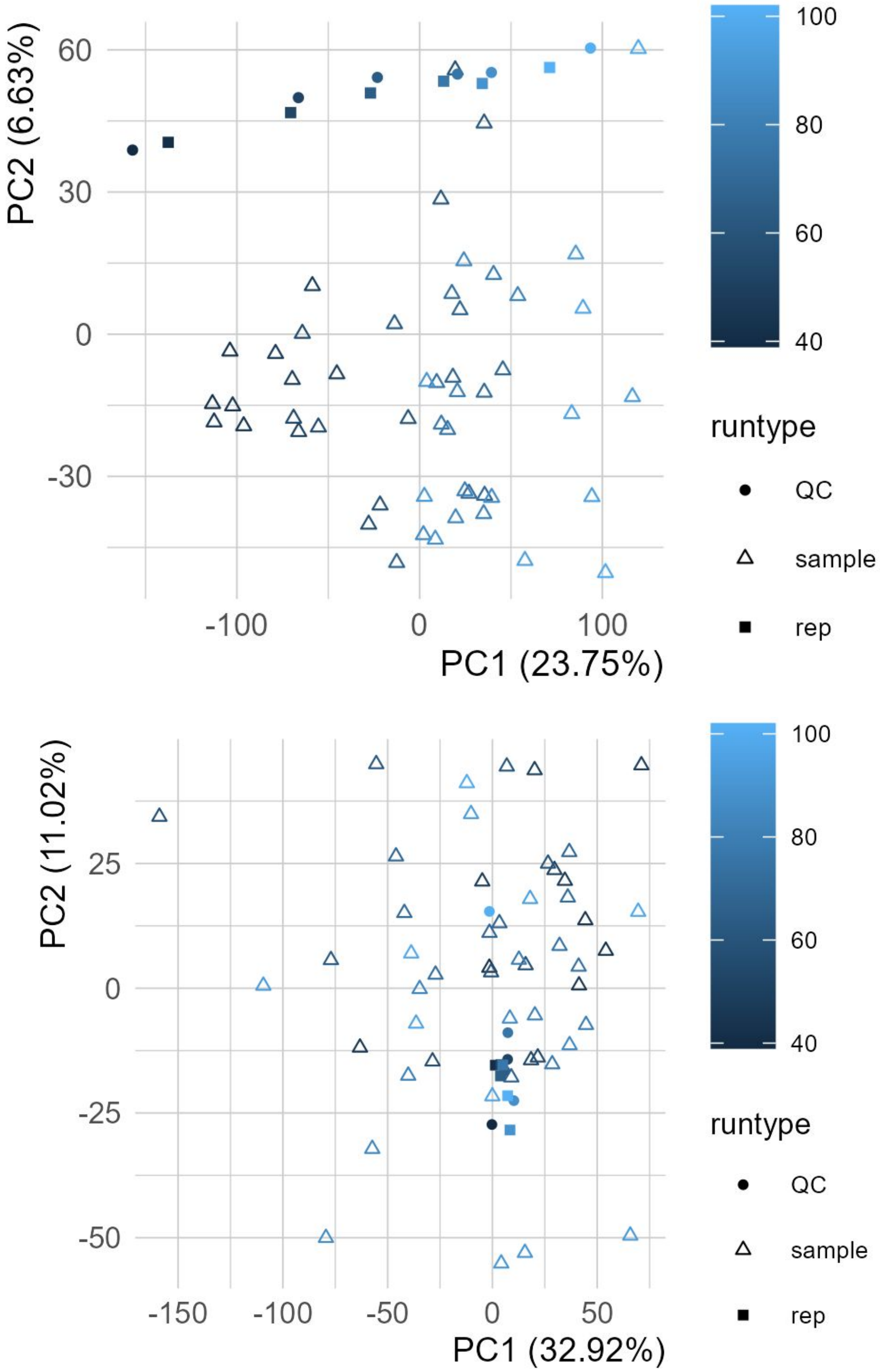

Figure S9. *Upper panel: PCA score plot of non-normalized measured areas of untargeted features in the ESCCAPE analysis. The run type is denoted by the symbols: circle (QC), triangle (sample) and square (technical replicate). The blue gradient indicates the injection number. A signal trend depending on injection order from the beginning the to end of the analysis can be observed. Lower panel: PCA score plot of lomec normalized measured areas of untargeted features in the ESCCAPE analysis. The run type is denoted by the symbols: circle (QC), triangle (sample) and square (technical replicate). The blue gradient indicates the injection number. The score plot shows that the trend based on injection order is successfully removed after normalization.*

Table S3. *Significant (adjusted P-value < 0.05) paired pairwise comparisons of RSD* of ISTD target peaks in the ESCCAPE analysis.*

group1	group2	statistic	p.value	p.adjust.method	test
FBSC-B	LMBSC	3.89711	0.01273	Holm	Durbin-Conover
FBSC-B	QC-RLSC	4.33013	0.00685	Holm	Durbin-Conover
FBSC-B	lomec	3.46410	0.01873	Holm	Durbin-Conover
LMBSC	QC-RLSC	8.22724	0.00004	Holm	Durbin-Conover
TIC	FBSC-B	8.66025	0.00003	Holm	Durbin-Conover
TIC	LMBSC	4.76314	0.00416	Holm	Durbin-Conover
TIC	QC-RLSC	12.99038	0.00000	Holm	Durbin-Conover
TIC	lomec	12.12436	0.00000	Holm	Durbin-Conover
w	FBSC-B	3.89711	0.01273	Holm	Durbin-Conover
non-normalized	QC-RLSC	8.22724	0.00004	Holm	Durbin-Conover
non-normalized	TIC	4.76314	0.00416	Holm	Durbin-Conover
non-normalized	lomec	7.36122	0.00011	Holm	Durbin-Conover
non-normalized	median	5.62917	0.00122	Holm	Durbin-Conover
lomec	LMBSC	7.36122	0.00011	Holm	Durbin-Conover
median	FBSC-B	9.52628	0.00001	Holm	Durbin-Conover
median	LMBSC	5.62917	0.00122	Holm	Durbin-Conover
median	QC-RLSC	13.85641	0.00000	Holm	Durbin-Conover
median	lomec	12.99038	0.00000	Holm	Durbin-Conover

Table S4. *Significant (adjusted P-value < 0.05) paired pairwise comparisons of RSD* of ISTD target peaks in the ENVIRONAGE analysis.*

group1	group2	statistic	p.value	p.adjust.method	test
FBSC-B	QC-RLSC	3.83406	0.02616	Holm	Durbin-Conover
LMBSC	QC-RLSC	4.92950	0.00557	Holm	Durbin-Conover
TIC	FBSC-B	3.83406	0.02616	Holm	Durbin-Conover
TIC	QC-RLSC	7.66812	0.00011	Holm	Durbin-Conover
TIC	lomec	7.12039	0.00022	Holm	Durbin-Conover
non-normalized	QC-RLSC	4.38178	0.01251	Holm	Durbin-Conover
non-normalized	lomec	3.83406	0.02616	Holm	Durbin-Conover
non-normalized	median	4.92950	0.00557	Holm	Durbin-Conover
lomec	LMBSC	4.38178	0.01251	Holm	Durbin-Conover
median	FBSC-B	5.47723	0.00240	Holm	Durbin-Conover
median	LMBSC	4.38178	0.01251	Holm	Durbin-Conover
median	QC-RLSC	9.31128	0.00002	Holm	Durbin-Conover
median	lomec	8.76356	0.00003	Holm	Durbin-Conover

Table S5. *Number of features with RSD* lower than 0.2 in the ESCCAPE dataset for the different normalization methods.*

Method	# feat with RSD* < 0.2
Non-normalized	6342
TIC	5391
Median	4818
FBSC-B	7623
Lomec	8288
LMBSC	6329
QC-RLSC	8329

Table S6. *Number of features with RSD* lower than 0.3 in the ENVIRONAGE dataset for the different normalization methods.*

Method	# feat with RSD* < 0.3
Non-normalized	2403
TIC	2710
Median	2587
FBSC-B	1973
Lomec	2245
LMBSC	2555
QC-RLSC	2999

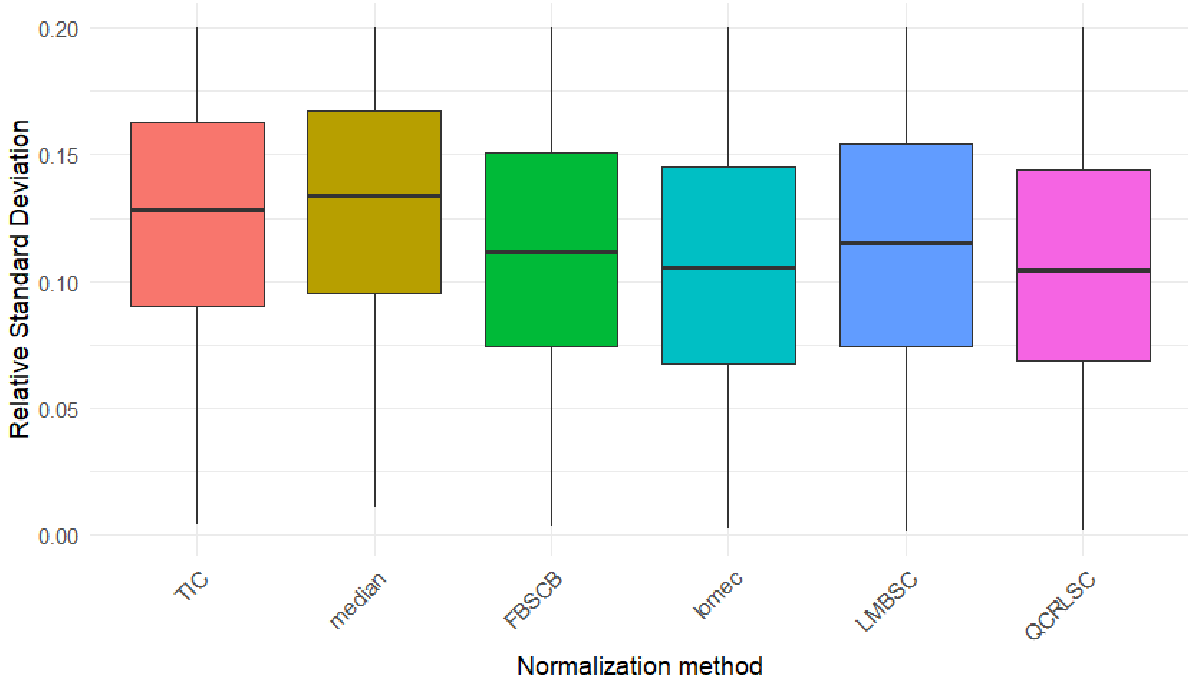

Figure S10. *Boxplots of the RSD* of features with RSD* < 0.2 in technical replicates of the ESCCAPE dataset.*

***Table S7**. Significant (adjusted P-value < 0.05) pairwise comparisons of RSD* of features with RSD* < 0.2 in the ESCCAPE analysis.*

group1	group2	statistic	p.value	p.adjust.method	test
FBSCB	LMBSC	2.42939	0.03025	Holm	Dunn
FBSCB	QCRLSC	7.25342	0.00000	Holm	Dunn
FBSCB	lomec	6.71979	0.00000	Holm	Dunn
LMBSC	QCRLSC	9.37630	0.00000	Holm	Dunn
TIC	FBSCB	14.66521	0.00000	Holm	Dunn
TIC	LMBSC	11.85880	0.00000	Holm	Dunn
TIC	QCRLSC	21.50693	0.00000	Holm	Dunn
TIC	lomec	21.00983	0.00000	Holm	Dunn
TIC	median	4.32248	0.00005	Holm	Dunn
lomec	LMBSC	8.86683	0.00000	Holm	Dunn
median	FBSCB	18.83576	0.00000	Holm	Dunn
median	LMBSC	15.97914	0.00000	Holm	Dunn
median	QCRLSC	25.50470	0.00000	Holm	Dunn
median	lomec	25.02167	0.00000	Holm	Dunn

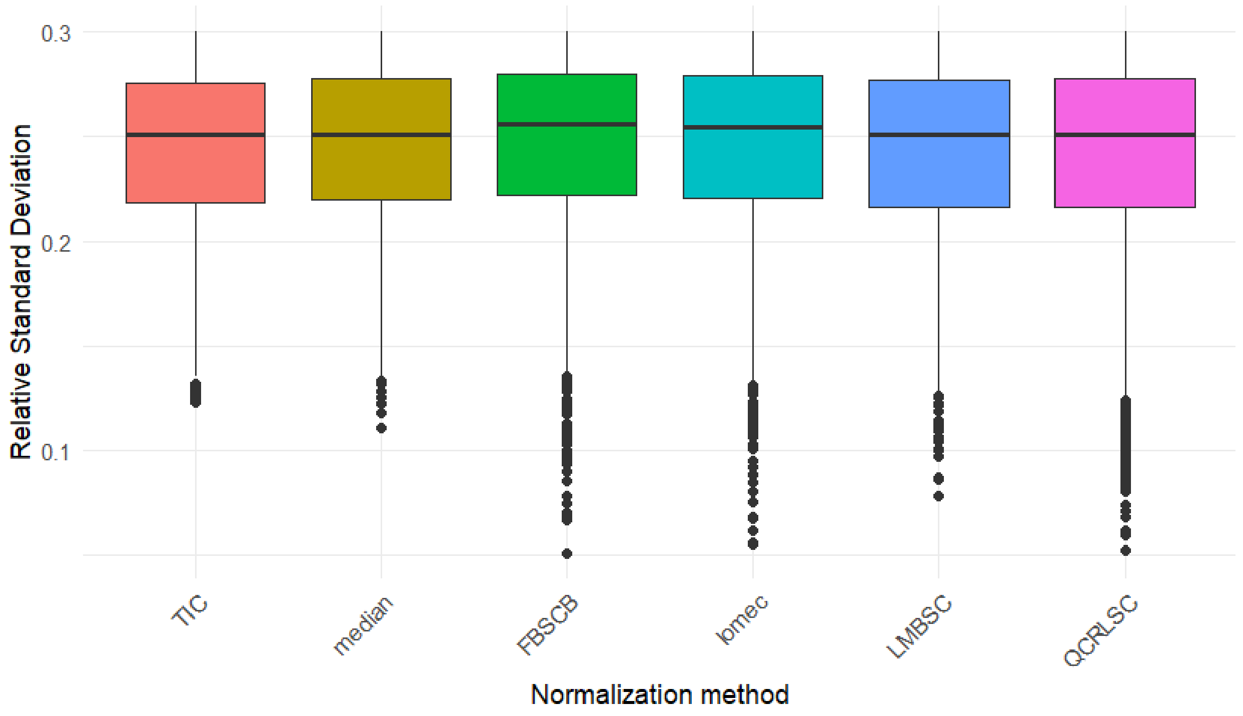

Figure S11. *Boxplots of the RSD* of features with RSD* < 0.3 in technical replicates of the ENVIRONAGE dataset.*

Table S8. *Significant (adjusted P-value < 0.05) pairwise comparisons of RSD* of features with RSD* < 0.3 in the ENVIRONAGE analysis.*

group1	group2	statistic	p.value	p.adjust.method	test
FBSCB	LMBSC	3.14562	0.02320	Holm	Dunn
FBSCB	QCRLSC	3.39189	0.01041	Holm	Dunn
TIC	FBSCB	2.95826	0.04022	Holm	Dunn

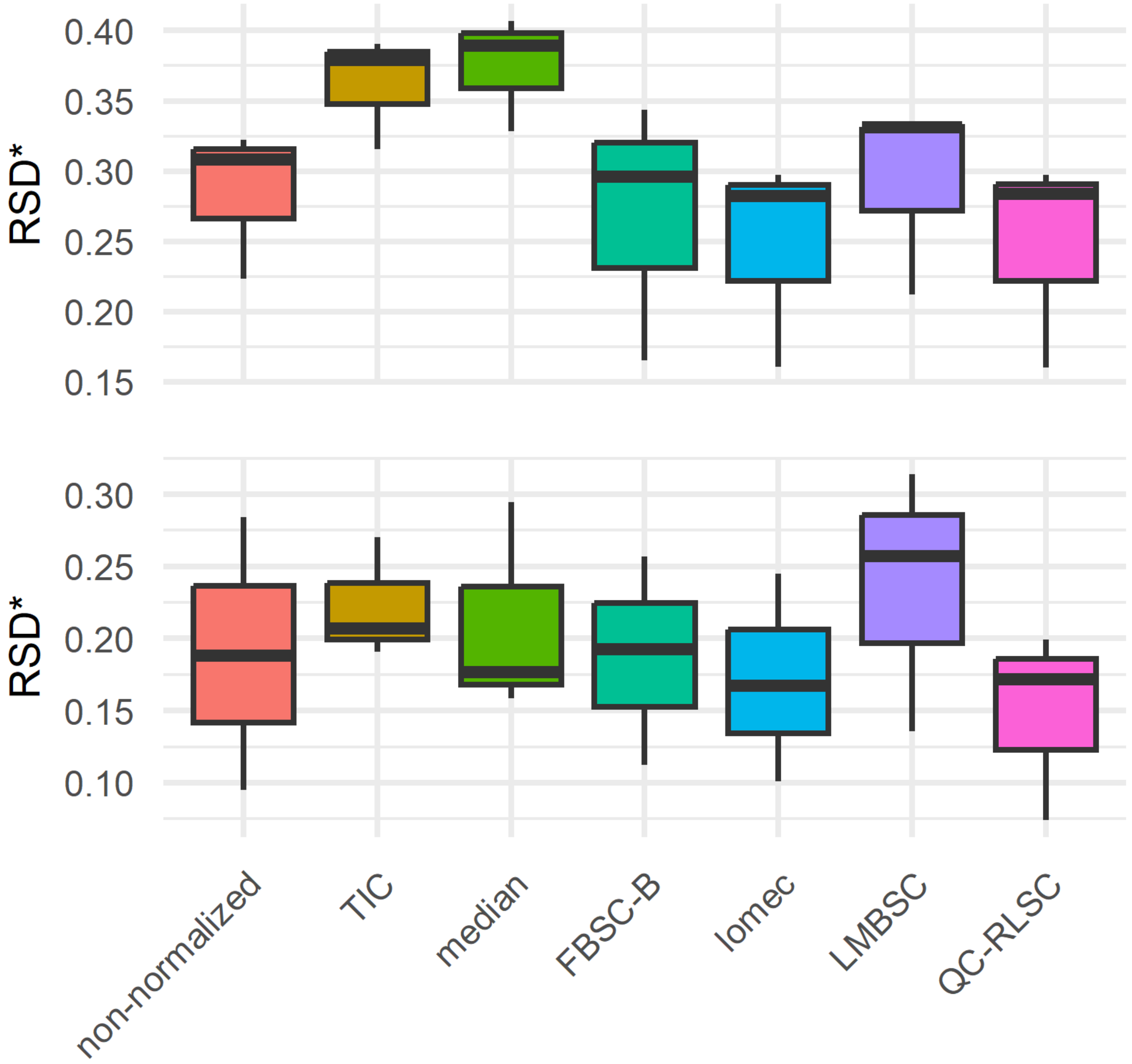

Figure S12. *Boxplots of RSD* of ISTD targets in sample runs (upper panel) and technical replicates (lower panel) in the ENVIRONAGE dataset. Sample-based methods (TIC and median) inflate RSD* more in sample runs compared to in technical replicates.*

Table S9. *Number of features with D-ratio lower than 0.4 in the ESCCAPE dataset for the different normalization methods.*

**Method**	**# feat with D-ratio < 0.4**
Non-normalized	3043
TIC	3627
Median	3019
FBSC-B	4142
Lomec	4125
LMBSC	3043
QC-RLSC	4036

Table S10. *Number of features with D-ratio lower than 0.5 in the ENVIRONAGE dataset for the different normalization methods.*

**Method**	**# feat with D-ratio < 0.5**
Non-normalized	688
TIC	1220
Median	943
FBSC-B	1017
Lomec	858
LMBSC	668
QC-RLSC	844

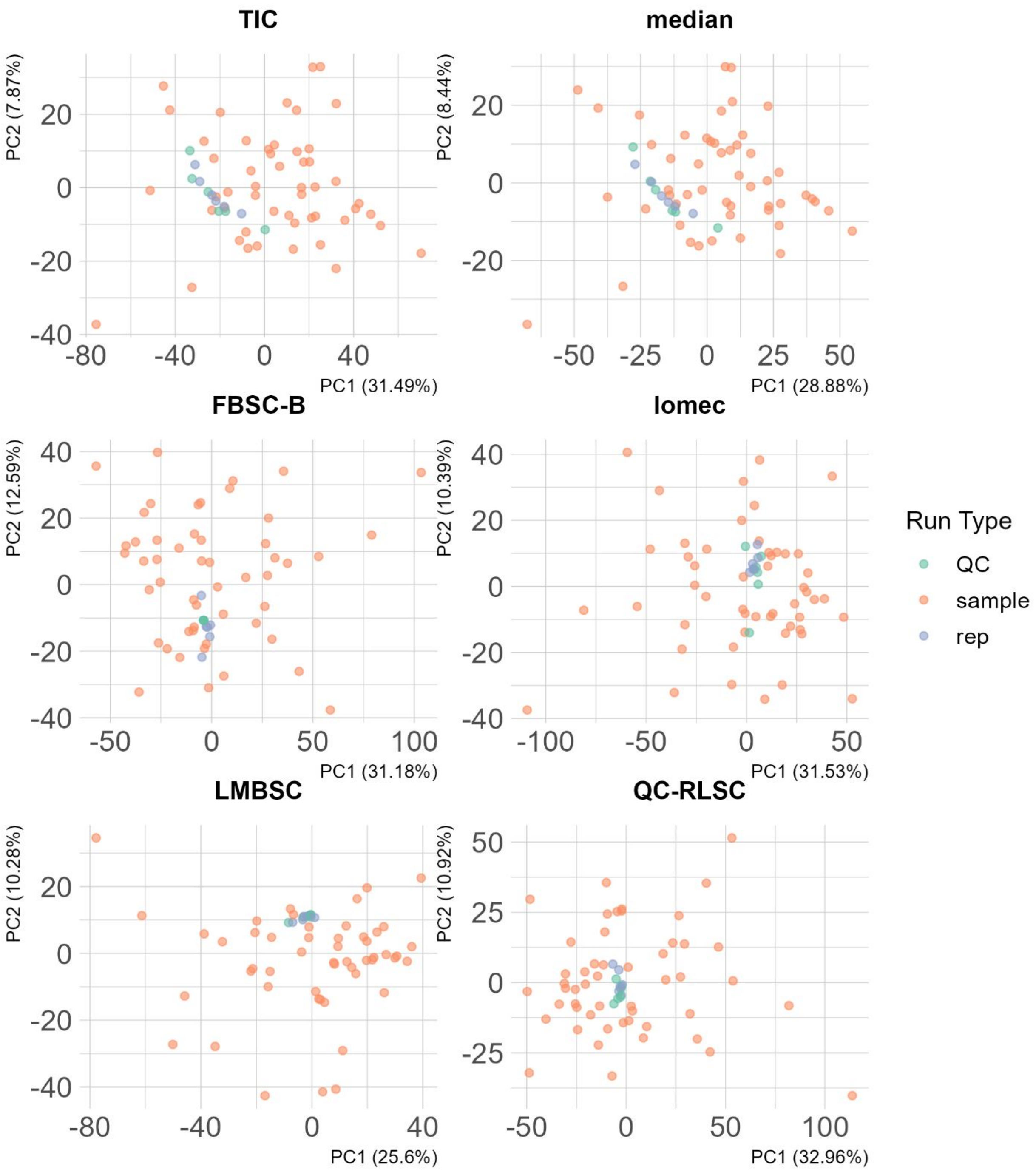

Figure S13. *PCA score plots of the ESCCAPE analysis. Each panel is the score plot based on the untargeted features retained after filtering out features with RSD* > 0.2 and D-ratio > 0.4.*

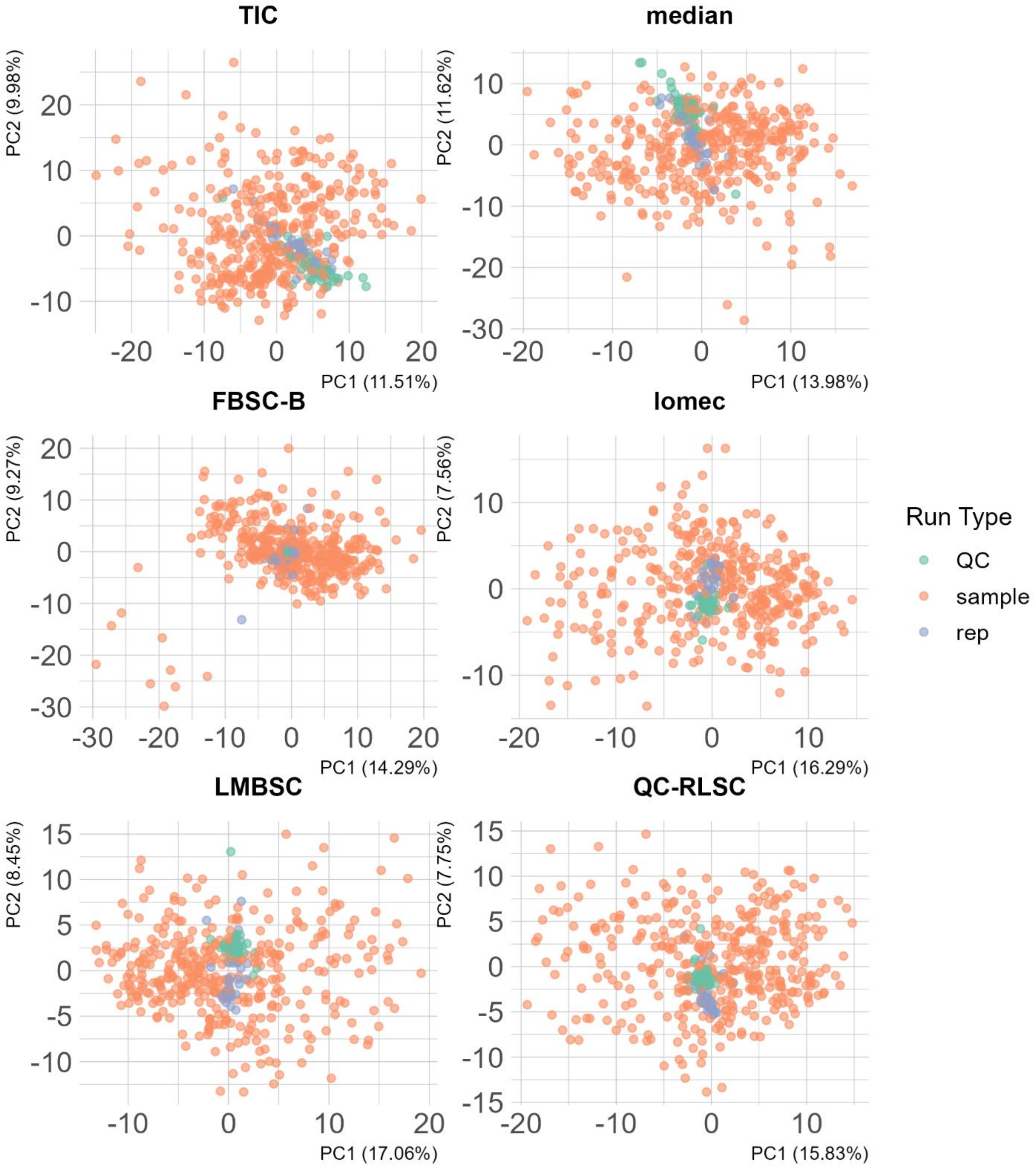

Figure S14 . *PCA score plots of the ENVIRONAGE analysis. Each panel is the score plot based on the untargeted features retained after filtering out features with RSD* > 0.3 and D-ratio > 0.5.*

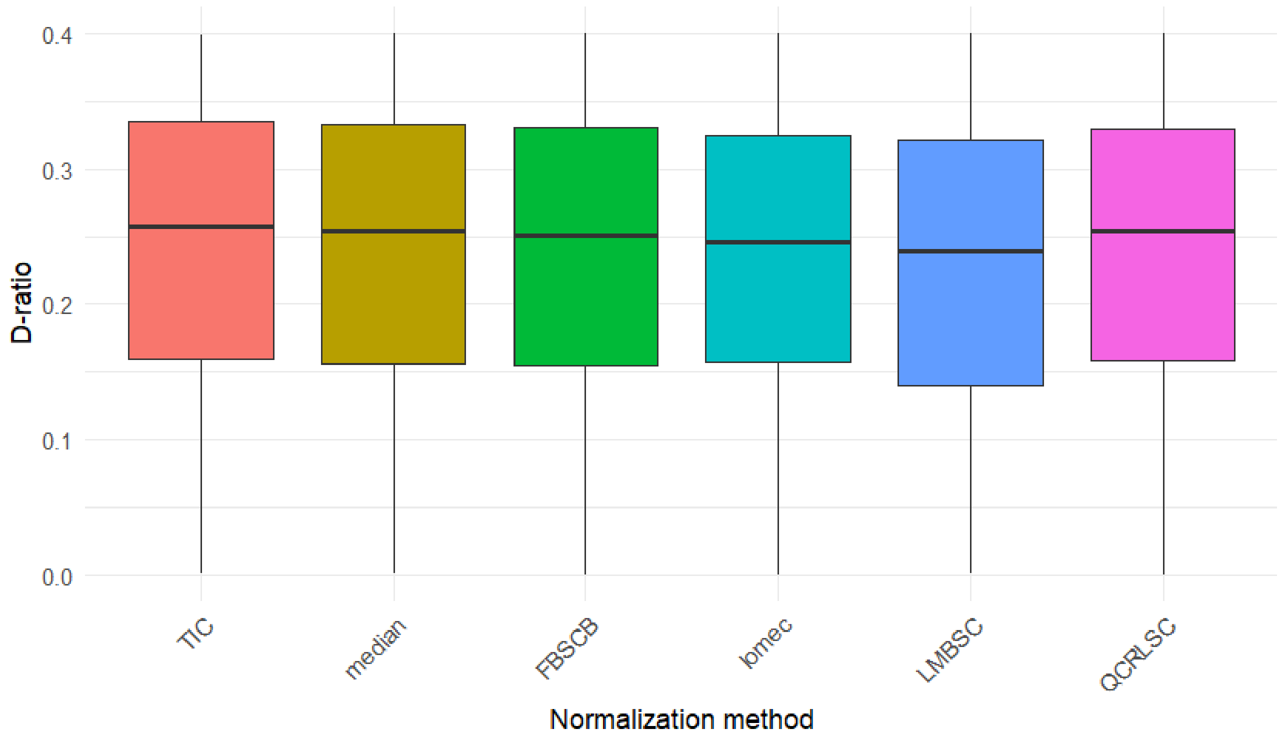

Figure S15. *Boxplots of the D-ratio of features with D-ratio < 0.4 in the ESCCAPE dataset.*

Table S11*. Significant (adjusted P-value < 0.05) pairwise comparisons of D-ratio of features with D-ratio < 0.4 in the ESCCAPE analysis.*

group1	group2	statistic	p.value	p.adjust.method	test
FBSCB	LMBSC	3.79222	0.00179	Holm	Dunn
LMBSC	QCRLSC	4.18474	0.00040	Holm	Dunn
TIC	LMBSC	4.68527	0.00004	Holm	Dunn
median	LMBSC	4.04238	0.00069	Holm	Dunn

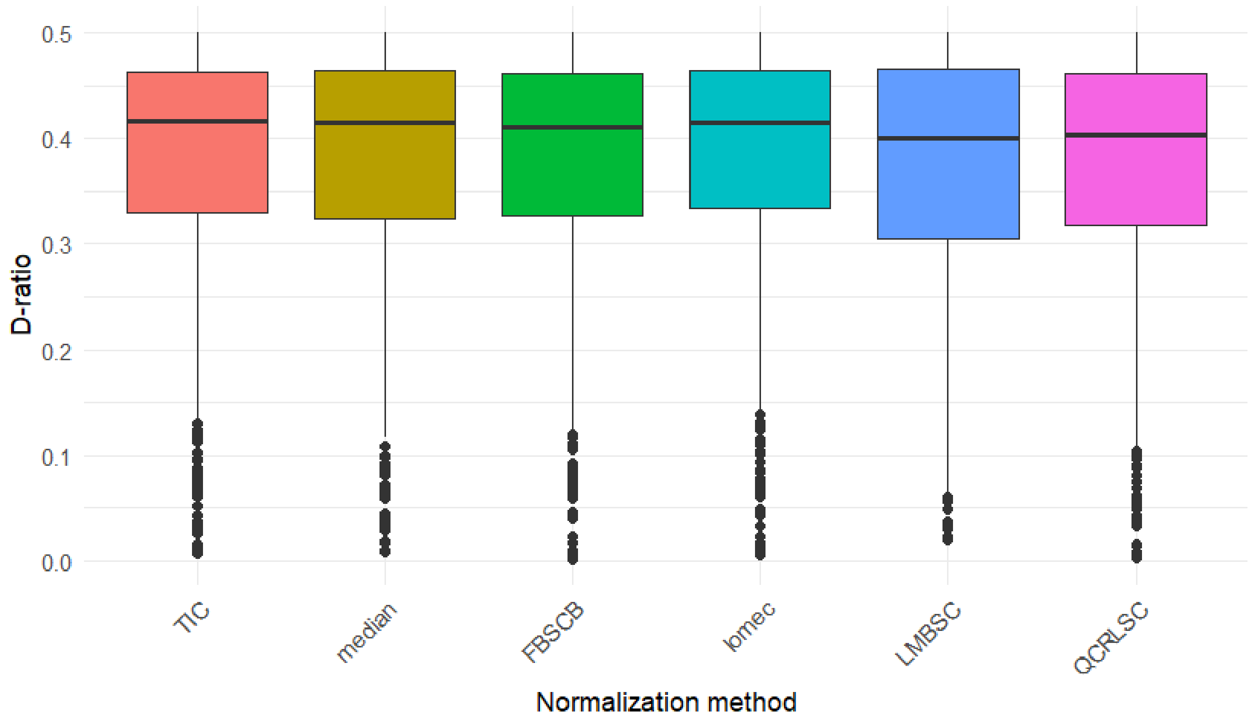

Figure S16. *Boxplots of the D-ratio of features with D-ratio < 0.5 in the ENVIRONAGE dataset.*

Table S12. *Number of features with an RSD* < 0.2 in technical replicates and a D-ratio < 0.4 in the ESCCAPE dataset.*

**Method**	**# feat**
TIC	2432
Median	1899
FBSC-B	2714
Lomec	2819
LMBSC	2127
QC-RLSC	2736

Table S13. *Number of features with an RSD* < 0.3 in technical replicates and a D-ratio < 0.5 in the ENVIRONAGE dataset.*

**Method**	**# feat**
TIC	435
Median	314
FBSC-B	323
Lomec	261
LMBSC	240
QC-RLSC	292

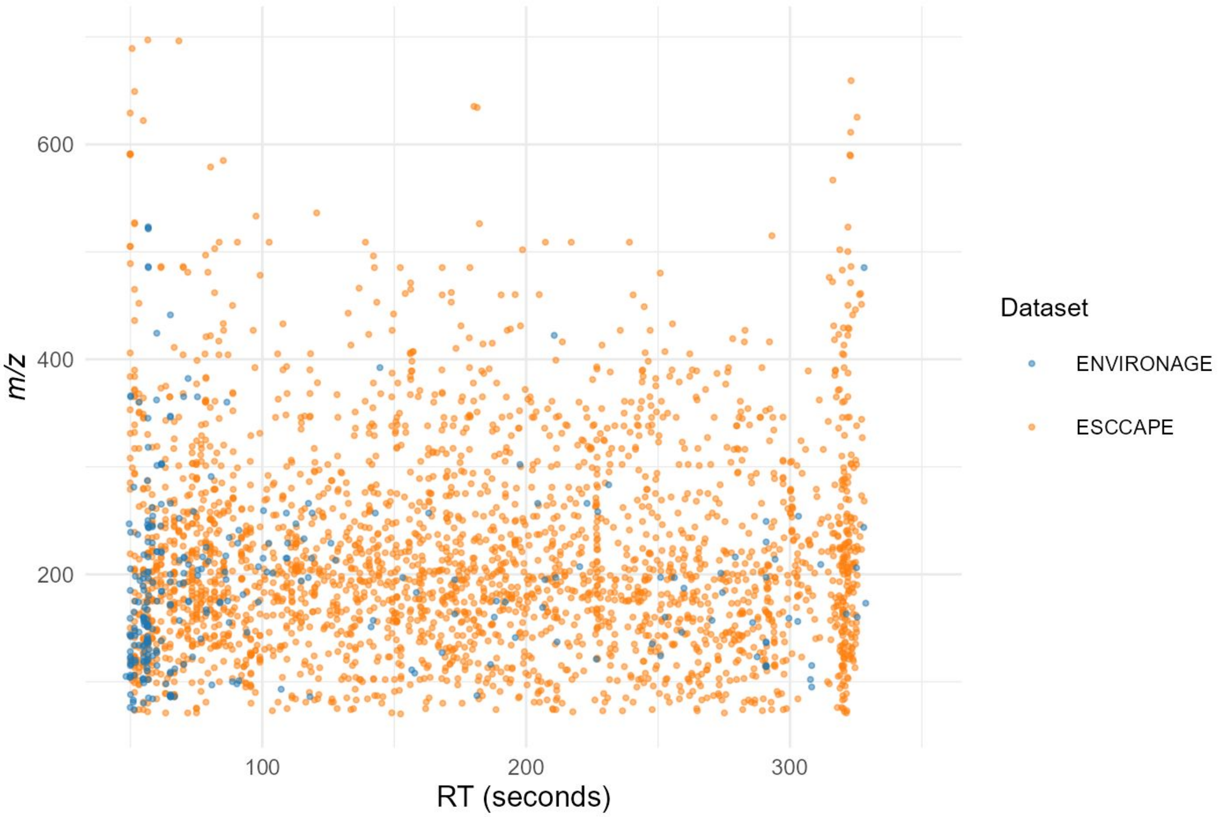

Figure S17. *DNA adductome map displaying features for ESCCAPE (orange) and ENVIRONAGE (blue) after respectively lomec and QC-RLSC normalization and feature filtering based on RSD and D-ratio.*
